# Supplementary material for: Postoperative surveillance and long-term outcome after endovascular aortic aneurysm repair in the Netherlands: study protocol for the retrospective ODYSSEUS study
Source: BMJ Open. 2020 Feb 18;10(2):e033584. doi: 10.1136/bmjopen-2019-033584 (PMC7045090; doi:10.1136/bmjopen-2019-033584)
Supplement: Author's manuscript [file bmjopen-2019-033584.draft_revisions.pdf]

BMJ Open is committed to open peer review. As part of this commitment we make the peer review history of every article we publish publicly available.

When an article is published we post the peer reviewers' comments and the authors' responses online. We also post the versions of the paper that were used during peer review. These are the versions that the peer review comments apply to.

The versions of the paper that follow are the versions that were submitted during the peer review process. They are not the versions of record or the final published versions. They should not be cited or distributed as the published version of this manuscript.

BMJ Open is an open access journal and the full, final, typeset and author-corrected version of record of the manuscript is available on our site with no access controls, subscription charges or pay-per-view fees (http://bmjopen.bmj.com).

If you have any questions on BMJ Open's open peer review process please email info.bmjopen@bmj.com

BMJ Open

Postoperative surveillance and long-term outcome after endovascular aortic aneurysm repair in the Netherlands: study protocol for the retrospective ODYSSEUS study.

Journal:	*BMJ Open*
Manuscript ID	bmjopen-2019-033584
Article Type:	Protocol
Date Submitted by the Author:	13-Aug-2019
Complete List of Authors:	Geraedts, Anna; Amsterdam UMC - Locatie AMC,<br>de Mik, Sylvana<br>Ubbink, Dirk<br>Koelemay, Mark<br>Balm, Ron
Keywords:	VASCULAR SURGERY, Aortic Aneurysm, Abdominal, Endovascular Procedures

SCHOLARONE™
Manuscripts

I, the Submitting Author has the right to grant and does grant on behalf of all authors of the Work (as defined in the below author licence), an exclusive licence and/or a non-exclusive licence for contributions from authors who are: i) UK Crown employees; ii) where BMJ has agreed a CC-BY licence shall apply, and/or iii) in accordance with the terms applicable for US Federal Government officers or employees acting as part of their official duties; on a worldwide, perpetual, irrevocable, royalty-free basis to BMJ Publishing Group Ltd ("BMJ") its licensees and where the relevant Journal is co-owned by BMJ to the co-owners of the Journal, to publish the Work in this journal and any other BMJ products and to exploit all rights, as set out in our licence.

The Submitting Author accepts and understands that any supply made under these terms is made by BMJ to the Submitting Author unless you are acting as an employee on behalf of your employer or a postgraduate student of an affiliated institution which is paying any applicable article publishing charge ("APC") for Open Access articles. Where the Submitting Author wishes to make the Work available on an Open Access basis (and intends to pay the relevant APC), the terms of reuse of such Open Access shall be governed by a Creative Commons licence – details of these licences and which Creative Commons licence will apply to this Work are set out in our licence referred to above.

Other than as permitted in any relevant BMJ Author's Self Archiving Policies, I confirm this Work has not been accepted for publication elsewhere, is not being considered for publication elsewhere and does not duplicate material already published. I confirm all authors consent to publication of this Work and authorise the granting of this licence.

For peer review only

Postoperative surveillance and long-term outcome after endovascular aortic aneurysm repair in the Netherlands: study protocol for the retrospective ODYSSEUS study.

A.C.M. Geraedts[1], S.M.L. de Mik[1], D.T. Ubbink[1], M.W.J. Koelemay[1], R. Balm[1], on behalf of the ODYSSEUS study group[2].

[1] Amsterdam University Medical Centres, University of Amsterdam, Amsterdam Cardiovascular Sciences, Department of Surgery, Meibergdreef 9, 1105AZ Amsterdam, The Netherlands

[2] Collaborator study group (names of the collaborators are found in the appendix): ODYSSEUS study group.

Contact details for corresponding author and reprints:

A.C.M. Geraedts, MD

Address: Amsterdam University Medical Centres, University of Amsterdam, Amsterdam Cardiovascular Sciences, Department of Surgery, Meibergdreef 9, 1105AZ Amsterdam, The Netherlands

Phone: +31(0)205662971

E-mail: a.c.geraedts@amsterdamumc.nl

Word count: 3102

Keywords:

Vascular Surgical Procedures; Aortic Aneurysm, Abdominal; Endovascular Procedures**.**

For peer review only

ABSTRACT

Introduction: Strict imaging surveillance protocols to detect complications following Endovascular Aneurysm Repair (EVAR) are common practice. However, controversy exists as to whether all EVAR patients need intense surveillance. The 2019 European Society for Vascular Surgery (ESVS) guidelines for management of abdominal aortic aneurysm (AAA) suggest that patients may be considered for limited follow-up with imaging if classified as 'low risk' for complications based on their initial postoperative imaging. The current study aims to investigate the intervention-free survival and overall survival stratified for patients with and without yearly imaging surveillance.

Methods and analysis: The ODYSSEUS study comprises a national multicentre retrospective cohort study in 17 medical centres. Consecutive patients with an asymptomatic or symptomatic infrarenal AAA who underwent EVAR between January 2007 and January 2012 will be included in this study with follow-up until December 2018. Clinical variables and all follow-up information will be retrieved in extensive data collection from the patient's medical records. In addition, an e-survey was sent to vascular surgeons at the 17 participating centres to gauge their opinions regarding the possibility of safely reducing the frequency of imaging surveillance. Primary endpoints are intervention after EVAR and aneurysm related mortality. The initial estimated sample size is 1997 patients.

Ethics and dissemination: The study has been approved by the Medical Ethics Review Committee of the Amsterdam UMC, location Academic Medical Centre, Amsterdam, the Netherlands. Study findings will be disseminated via presentations at conferences and publications in peer-reviewed journal.

Registration: Netherlands Trial Registry, NL6953 (old: NTR28773). Registered 5 Apr 2018. URL: https://www.trialregister.nl/trial/6953

For peer review only

ARTICLE SUMMARY

Strengths and limitations of this study

- The main strength of this study is that it can accumulate data from large number of patients with long-term follow-up up to 11 years and that it captures all surveillance visits, long-term outcomes and mortality post-EVAR.
- The sample size will be large enough to enable survival and regression analyses in small sub-groups of patients.
- The main limitation of the study is due to the nature of administrative data, it allows only the collection of data that was documented in the patient medical records.

For peer review only

INTRODUCTION

Endovascular Aneurysm Repair (EVAR) of the abdominal aorta has become the primary treatment of patients with an abdominal aortic aneurysm (AAA).[1] Both the Society for Vascular Surgery (SVS) International Guidelines and the instructions for use (IFU) of endograft manufacturers recommend yearly imaging surveillance for all patients after EVAR.[2] However, if the patient is classified as 'low risk' for complications based on initial post-operative imaging, the 2019 European Society for Vascular Surgery (ESVS) guidelines recommend delaying imaging until five years after repair.[3] This movement towards reducing the imaging frequency will benefit patients, medical centres and health care costs.

Imaging surveillance by Computed Tomography Angiography (CTA) may increase the attributable lifetime cancer risk of patients, as well as putting them at risk of developing nephropathy due to contrast exposure. If yearly CTA is replaced by duplex ultrasonography (DUS) patients still experience the burden of additional hospital visits. Moreover, compliance with yearly imaging is suboptimal and non-adherence to yearly imaging does not appear to be associated with poorer outcomes.[4][5]

It has been questioned whether yearly imaging is necessary for all EVAR patients, and if a specific group of patients can be identified for which surveillance intervals can safely be extended, as is suggested by the new guideline.[3] For these reasons, in the Netherlands the Observing a Decade of Yearly Standardised Surveillance in EVAR patients with Ultrasound or CT Scan (ODYSSEUS) study has been designed. In this study of approximately 2000 patients with 6-11 years of follow-up, we aim to determine when, and in which patients, it is safe to deviate from the current annual surveillance protocols.

For peer review only

Background and relevant literature and data

Before initiating the ODYSSEUS study, we conducted a survey among Dutch vascular surgeons to find out if they support the possibility of reducing the frequency of imaging surveillance. In this survey, vascular surgeons reported the main reasons patients did not comply with follow-up visits, i.e. they had forgotten the appointment or were prevented by force majeure. Most physicians estimated that less than 10% of their patients had missed one or more follow-up visits post-EVAR. This might be an overestimation of the true adherence to follow-up visits, as these observations are in contrast with a study reporting that only 43% of patients had complete surveillance.[4]

We also asked participating vascular surgeons to upload their standard post-EVAR protocol to investigate if there were differences between centres in the Netherlands. In all centres imaging took place within the first 3 months after surgery, mostly by CTA. Most centres comply with their own post-EVAR surveillance protocols, which have many commonalities with the SVS and ESVS guidelines. Only one centre utilizes precisely the same post-EVAR surveillance protocol as recommended by the SVS guidelines. Another centre had already reduced follow-up imaging to once every five years, using either CTA or DUS as is stated in the new ESVS guidelines.[3] While vascular surgeons still seem to adhere to their hospital-specific protocol, they do support the need for reducing follow-up by selecting a group of patients for which yearly follow-up can safely be omitted. However, some surgeons indicated that more evidence is needed than is available in the current literature.[6][7]

In studies that have investigated the indications for post-EVAR intervention, it is stated that 61-98% of interventions were necessary because of symptoms and not because of findings at surveillance imaging. This suggests that post-EVAR surveillance protocols provide no benefit to a large group of patients, as complications occur in between surveillance visits.[8][9] Imaging surveillance may even lead to unnecessary interventions and it does not appear to be associated with improved survival.[9][10] We hypothesize that the requirement

For peer review only

for routine imaging for patients at low risk can be reduced. However, novel endovascular devices still require more intensive surveillance as the short- and long-term results of those devices remain undetermined.

For peer review only

STUDY OBJECTIVES

The objective of this study is to evaluate whether imaging surveillance frequency can be safely reduced in a selected group of EVAR patients, for example in patients with an asymptomatic or symptomatic infrarenal AAA who underwent EVAR and who had no abnormalities on the first postoperative CTA. The clinical course of a large cohort of patients will be evaluated with follow-up ranging between 6 and 11 years. Baseline patient characteristics, aortic anatomy and details of the operation will be derived from the patient's medical record. The first milestone during follow-up is the first postoperative CTA. This scan either shows complications such as endoleaks, malposition or migration of the graft, or the absence thereof. All follow-up visits, imaging studies, as well as all interventions after EVAR and outcomes will be registered. Our hypothesis is that patients with less follow-up will have better outcomes regarding the number of interventions and aneurysm related mortality compared to patients with annual follow-up. Regarding the intervention rates, it is expected that adherence to imaging surveillance may detect more abnormalities triggering re-interventions, which in itself may cause additional complications and perhaps even a decrease in survival rates. We hypothesize that the need for routine imaging for patients with no abnormalities at their initial CTA can be decreased.

For peer review only

METHODS AND ANALYSIS

The study protocol has been designed according to the Standard Protocol Items: Recommendations for Interventional Trials (SPIRIT) statement and the CONsolidated Standards of Reporting Trials (CONSORT).[11,12] The study is registered in The Netherlands National Trial Registry as registration number NL6953**,** available at: https://www.trialregister.nl/trial/6953

General study design

A multicentre retrospective cohort study in 17 medical centres in the Netherlands. Data will be collected from the medical records of all consecutive patients with AAA who underwent EVAR between January 2007 and January 2012. This selection provides a length of follow-up of 6-11 years on December 2018. Patients will be divided into three groups: A) patients without abnormalities at their first postoperative CTA with yearly imaging surveillance, B) patients without abnormalities at their first postoperative CTA without yearly imaging surveillance and C) patients with abnormalities at their first postoperative CTA (Figure 1). This retrospective design has the advantage of collecting long term follow-up data. The Dutch Dream trial found that the number of interventions starts to rise 4 years after EVAR and the long-term results of the EVAR-1 trial show that EVAR has an early survival benefit but inferior late survival compared with open surgical repair.[13,14] This is in contrast to the recently published long-term results of the OVER trial in which no difference was observed between EVAR and OSR in the primary outcome of all-cause mortality.[15] Hence, a prospective study would take approximately 8 to 10 years to gather enough patients with adequate follow-up.

The standard of care is defined by the current guidelines and instructions for use (IFU). The usual follow-up schedule in the IFU is: CTA and abdominal X-ray at 30 days, 6 and 12 months and yearly thereafter. The guidelines from the European Society for Vascular Surgery

For peer review only

(2019) recommend a CTA 30 days after EVAR. If there is adequate seal and no endoleak patients are classified as low risk and CTA may take place 5 years later. If there is an inadequate seal and endoleak type I/III patients could be either evaluated for re-intervention or if sac shrinkage occurs yearly DUS is recommended. In the 2018 SVS guideline CTA at one and 12 months is recommended and if neither endoleak nor sac enlargement is documented, DUS is suggested for annual postoperative surveillance. In our study design the definition of compliance is undergoing imaging surveillance every 16 months since patients in most centres will be rescheduled if they missed their annual follow-up visit.

Study population

Patients eligible for this retrospective study are all adults who underwent elective EVAR for asymptomatic or symptomatic infrarenal AAA between January 2007 and January 2012. Table 1 gives a more detailed overview of the inclusion and exclusion criteria.

Patient and public involvement

No patients were involved in the research design and conception of this research study.

Date range of the study

Data will be extracted from patient medical records retrospectively and entered into a database with data validation from December 2018 until June 2020.

Subject selection

A retrospective cohort study of consecutive patients treated at 17 vascular centres is to be performed. All patients are eligible and the opt-out procedure will be used to allow patients to object to participation within four weeks, which is in accordance with the Dutch Code of Civil Procedure. The Medical Ethics Review Committee of the Amsterdam UMC, location Academic Medical Centre, Amsterdam, has confirmed that the Medical Research Involving

For peer review only

Human Subjects Act (WMO) does not apply to our study. This study is conducted according to the General Data Protection Regulation (AVG 2016) and the Medical Treatment Agreement Act (WGBO).

Data sources

Paper or electronic medical records are used in order to identify participants who match study-defined criteria.

Primary and secondary endpoints

The primary outcome parameters of this study are interventions and aneurysm related mortality for patients who had a normal initial postoperative CTA and who do adhere to our definition of yearly imaging surveillance over a 6 to 11-year follow-up period, compared to those who do not adhere to our definition.

Interventions are EVAR-related interventions defined by the SVS reporting standards as postoperative adjunctive manoeuvres.[16][17] Interventions for wound complications at the access site are not included, since these are detectable without the use of imaging.

Date of death during follow-up, if applicable, will be obtained from patient medical records and verified by the Dutch municipal personal records database (GBA).

Details of all surveillance imaging are obtained from patient medical records and radiology reports. The time between imaging appointments is calculated to determine whether patients adhere to this studies definition of with yearly imaging surveillance, i.e. within every 16 months.

Date, type, indication and outcome of all postoperative imaging during follow-up are obtained from patient medical records, specifically imaging order forms and radiology reports. A normal initial postoperative CT scan is defined as a CT scan which shows no endoleaks, endograft migration (>10 mm), kinking or obstruction. All imaging outcomes are

For peer review only

based on the report compiled by radiologists. These reports will not be re-evaluated by an independent radiologist, since we want to base our outcomes on real life data.

Other secondary outcomes are all-cause mortality, the registration of type I, type II, type III and type IV endoleak, graft or outflow (iliac) occlusion, aneurysm rupture and endograft infection. This is also obtained from patient medical records, specifically radiology reports and Dutch municipal personal records database (GBA).

Date of aneurysm rupture is obtained from patient medical records, specifically operative reports, radiology reports and progress notes.

Costs of all EVAR-related imaging and outpatient clinic visits will be calculated per patient. Cost is defined as volume times price. Prices from the "Cost manual of the Dutch Health Care Institute" will be used. Costs for the patients will also be included. The quality-adjusted life years (QALYs), a generic measure of disease burden including both the quality and quantity of life, cannot be calculated with this retrospective design.

Sample size and power

Sample size calculation for this study is based on a comparison of the number of interventions (proportions) in two groups (patients undergoing yearly standardised imaging surveillance versus patients not undergoing standardised imaging surveillance). We use a superiority design. To have a 90% chance of detecting, as significant at the 5% level, an increase in intervention-free rate from 75% after 7 years in the surveillance group and 82% in the nonstandard-surveillance group. This results in 719 patient per group and 1438 patients in total. Since the first CTA of approximately 20% of patients is abnormal (1438/0.8=), 1798 patients are needed. In addition, we expect incomplete data in 10% of the patients which results in a minimum of (1798/0.9=), 1997 patients are needed in total. With this number of patients we can also calculate a 3% difference in aneurysm related mortality. We chose a one-sided significance level (non-inferiority) of 0.050 and for standard

For peer review only

proportion a 95% non-aneurysm related mortality and thus 5% freedom from aneurysm-related mortality after 7 years: 0.950. For equivalence limit difference, we chose an acceptable difference between groups of 3%, in which if differences in aneurysm related mortality equals 3%, they are considered non-inferior. Test-expected proportion is then equal to the standard proportion 0.97. Thus, the expected difference is 0, calculated with a power of 80%. This results in 653 patients per group and 1306 patients in total. Since the first CTA of approximately 20% of patients is abnormal (1306/0.8=), 1632 patients are needed. In addition, we expect incomplete data in 10% of the patients which results in a minimum of (1632/0.9 =), 1813 patients are needed in total.

Statistical methods

Differences in baseline characteristics between patients undergoing or not undergoing yearly standardised imaging surveillance by either CTA or DUS, will be analysed using the Chi-square or Fisher's exact test for categorical variables and the student's t-test or Mann Whitney test for continuous variables, if appropriate.

The primary endpoints, i.e. freedom from intervention and survival will be estimated by Kaplan-Meier survival analysis and differences between groups will be assessed with the log-rank test.

Secondary endpoints such as freedom from aneurysm rupture between patients with and without yearly standardised imaging surveillance will be estimated by Kaplan-Meier survival analysis, and differences between these groups will be calculated with the log-rank test. In addition, the association between postoperative intervention and the following covariates: age, gender, AAA diameter, ASA classification, neck length (>15mm), neck angulation (>60°), type of graft, initial postoperative CTA with or without abnormalities will be investigated with multivariate Cox-regression analysis. All statistical analyses will be done

For peer review only

with SPSS software (IBM, version 25). The level for statistical significance is set at a p-value < 0.05.

For peer review only

DISCUSSION

The goal of this study is to evaluate whether a reduction in follow-up visits and imaging and thus costs, in patients operated on for an asymptomatic or symptomatic AAA with EVAR is safe. We hypothesize that there will be less interventions and no difference in aneurysm related mortality in patients with less intensive follow-up. With the results of this study we aim to provide scientific evidence helping vascular surgeons decide whether less vigilant follow-up after EVAR may be considered for patients classified in the low risk group.

The strengths of the ODYSSEUS study are that it can accumulate the data of a large number of consecutive EVAR patients with a follow-up of 6 to 11 years, and that it captures all surveillance visits, long-term outcomes and mortality post-EVAR. Moreover, 17 medical centres throughout the Netherlands are participating in this study, including university and general hospitals, thereby reducing selection bias.

An e-survey has been sent to all vascular surgeons participating in the ODYSSEUS study. This shows that yearly imaging surveillance is upheld by most vascular surgeons in the Netherlands. In addition, most physicians agree that yearly imaging frequency can be safely reduced in a specific group of EVAR patients. As support for this reduction in frequency is evident in the Netherlands, the next step will be to study the groups for which it will be safe to deviate from the widely accepted surveillance protocols.

Our study is subject to limitations due to the nature of administrative data. As with all studies using administrative data, it allows only the collection of data that was documented in patient medical records. The mentioned e-survey has only been sent to participating vascular surgeons, perhaps surgeons participating in the ODYSSEUS study strongly believe that imaging surveillance frequency can be reduced. This may have provided a biased view of post-EVAR follow-up in the Netherlands. However, most of the high-volume EVAR centres in the Netherlands have been included.

For peer review only

In conclusion, with the ODYSSEUS study we aim to confirm the follow-up protocol of the recent ESVS guideline delaying imaging after 5 years if classified in the low risk group and therefore aim to investigate the intervention-free-survival and aneurysm related mortality for patients with and without yearly imaging surveillance.

For peer review only

STUDY ADMINISTRATION

Ethics and dissemination

The Medical Ethics Review Committee of the Academic Medical Centre, Amsterdam, has reviewed and approved our study protocol version 1.6 dated 26 March 2018. The study is being conducted according to the principles of the Declaration of Helsinki in the current version of Fortaleza, Brazil (2013). Principles of good clinical practice will be respected. Study participation is voluntary.

Data management

All data obtained during the course of the study are considered to be confidential and will not be distributed to third parties. Patient data are stored anonymously under a code. Only the principal investigator or researchers authorised by the principal investigator have access to the key file.

Informed consent

For this retrospective study no informed consent is required. Patients are informed by the researchers that their patient information will be used for research. Patients are able to opt-out of their information being used by returning the opt-out form attached to the patient information letter via email or stamped return envelopes.

Funding statement

This study is funded by the AMC Foundation and with financial support of the Netherlands Organization for Health Research and Development (ZonMw; grant 843004119). The duration of the study is from July 15, 2018 to July 15, 2021. The AMC Foundation nor ZonMw is involved in the study design, writing of the manuscript or the decision to submit the manuscript for publication.

For peer review only

Conflict of interest

The authors declare that they have no competing interests.

Author contributions

Conception and design of study: A.C.M. Geraedts, S.M.L. de Mik, D.T. Ubbink, M.W.J. Koelemay, R. Balm.

Drafting the manuscript: A.C.M. Geraedts, S.M.L. de Mik.

Revising the manuscript critically for important intellectual content: D.T. Ubbink, M.W.J. Koelemay, R. Balm.

Approval of the version of the manuscript to be published: A.C.M. Geraedts, S.M.L. de Mik, D.T. Ubbink, M.W.J. Koelemay, R. Balm.

Agreement to be accountable for all aspects of the work: A.C.M. Geraedts, S.M.L. de Mik, D.T. Ubbink, M.W.J. Koelemay, R. Balm.

Data statement

Castor EDC, the Netherlands will be used for data collection and will be managed by qualified personnel. The data output can be opened in SPSS. Data is published via an DOI-code that will be requested via Figshare. Storage of the data under the DOI-code is one of the requirements of ZonMw.

For peer review only

REFERENCES

1.The EVAR trial participants. Comparison of endo vascular aneurysm repair with open repair in patients with abdominal aortic aneurysm (EVAR trial 1), 30-day operative mortality results: randomized controlled trial. Lancet 2004; 364: 843–48.

2. Chaikof EL, Brewster DC, Dalman RL, et al. SVS Practice guidelines for the care of patients with an abdominal aortic aneurysm: executive summary. J Vasc Surg 2018;67(1):2-77..

3. Wanhainen A, Verzini F, van Herzeele I, et al. European Society for Vascular Surgery (ESVS) 2019 Clinical Practice Guidelines on the Management of Abdominal Aorto-Iliac Artery Aneurysms. Eur J Vasc Endovasc Surg. 2019 Jan;57(1):8-93.

4. Garg T, Baker LC, Mell MW. Postoperative Surveillance and long-term outcomes after endovascular aneurysm repair among Medicare beneficiaries. JAMA Surg 2015;150:957-63.

5. Kret MR, Azarbal AF, Mitchell EL, et al. Compliance with long-term surveillance recommendations following endovascular aneurysm repair or type B aortic dissection. J Vasc Surg 2013 Jul;58(1):25-31.

6. Baderkhan H, Haller O, Wanhainen A, Björck M, Mani K. Follow-up after endovascular aortic aneurysm repair can be stratified based on first postoperative imaging. Br J Surg 2018;105:709-18

7. Bastos Goncalves F, Baderkhan H, Verhagen HJ, Wanhainen A, Björck M, Stolker RJ, et al. Early sac shrinkage predicts a low risk of late complications after endovascular aortic aneurysm repair. Br J Surg 2014;101:802-10.

8. Karthikesalingam A, Holt PJ, Hinchliffe RJ, et al. Risk of reintervention after endovascular aortic aneurysm repair. Br J Surg 2010;97(5):657-63.

9. Dias NV, Riva L, Ivancev K, et al. Is there a benefit of frequent CT follow-up after EVAR? Eur J Vasc Endovasc Surg 2009;37(4):425-30.

10. De Mik SML, Geraedts ACM, Ubbink DT, et al. Effect of imaging surveillance after Endovascular Aneurysm Repair on reinterventions and mortality: a systematic review and meta-analysis. J Endovasc Ther. 2019;26(4):531-541.

For peer review only

11.Chan AW, Tetzlaff JM, Gotzsche PC, Altman DG, Mann H, Berlin JA, et al. SPIRIT 2013 explanation and elaboration: guidance for protocols of clinical trials. BMJ. 2013;346.

12. Campbell MK, Piaggio G, Elbourne DR, Altman DG, Group C. Consort 2010 statement: extension to cluster randomised trials. BMJ. 2012;345.

13. De Bruin Jorg L, Baas Annette F, Buth Jaap, et al, for the DREAM Study Group. Long-term outcome of open or endovascular repair of abdominal aortic aneurysm. N Engl J Med 2010;362:1881e9.

14. Patel R, Powell JT, Sweeting MJ, et al. The UK EndoVascular Aneurysm Repair (EVAR) randomised controlled trials: long-term follow-up and cost-effectiveness analysis. Health Technol Assess. 2018 Jan;22(5):1-132.

15. Lederle FA, Kyriakides TC, Stroupe, KT, et al. Open versus endovascular repair of abdominal aortic aneurysm. N Engl J Med 2019;380(22):2126-2135.

16. Chaikof EL, Blankensteijn JD, Harris PL, et al. Reporting standards for endovascular aortic aneurysm repair. J Vasc Surg. 2002;35(5):1048-60.

17. Alonso J, Bartlett SJ, Rose M, et al. The case for an international patient-reported outcome measurement information system (PROMIS®) initiative. Health Qual life Outcomes 11:210, 2013.

For peer review only

Tables

Table 1

Retrospective cohort

Inclusion criteria	Exclusion criteria
Age ≥ 18 years<br>Patient with an (a)asymptomatic infrarenal abdominal aortic aneurysm<br>EVAR between January 2007 and January 2012<br>Patients with an initial postoperative CTA within 90 days after EVAR	Connective tissue disease<br>Patients who objected to their retrospective data being used

For peer review only

Figures

Figure 1. Patient subgroups

For peer review only

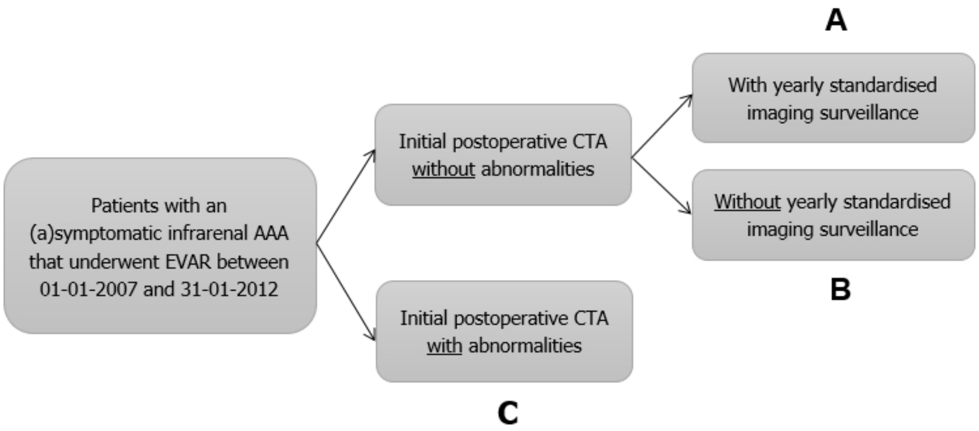

Figure 1. Patient subgroups

APPENDIX

COLLABORATORS TO THE ODYSSEUS STUDY GROUP (in alphabetical order):

R. Balm[1], J.W. Elshof[2], B.H.P. Elsman[3], J.F. Hamming[4], J.A. van Herwaarden[5], R.H.J. Kropman[6], M.M. Lensvelt[1], P.P. Poyck[7], G.W.H. Schurink[8], A.A.E.A de Smet[9], S.M. van Sterkenburg[10], C. Ünlü[11], A.C. Vahl[12], H.J.M. Verhagen[13], P.W.H.E. Vriens[14], J.P.P.M. de Vries[6,15], J.J. Wever[16], W. Wisselink[1], C.J. Zeebregts[15]

[1]Department of Surgery, Amsterdam University Medical Centres, Amsterdam Cardiovascular Sciences, Amsterdam, The Netherlands
[2]Department of Surgery, VieCuri Medical Centre, Venlo, The Netherlands
[3]Department of Surgery, Deventer Hospital, Deventer, The Netherlands
[4]Department of Surgery, Leiden University Medical Centre, Leiden, the Netherlands
[5]Department of Surgery, University Medical Centre Utrecht, Utrecht, The Netherlands
[6] Department of Surgery, St. Antonius Hospital, Nieuwegein, the Netherlands
[7]Department of Vascular and Transplant Surgery, Radboud University Medical Centre, Nijmegen, The Netherlands
[8]Department of Vascular Surgery, Maastricht University Medical Centre, Maastricht, The Netherlands
[9]Department of Surgery, Maasstad Hospital, Rotterdam, the Netherlands
[10]Department of Surgery, Rijnstate Hospital, Arnhem, the Netherlands
[11]Department of Surgery, Noordwest Ziekenhuisgroep, Alkmaar, the Netherlands
[12]Department of Surgery, OLVG, Amsterdam, The Netherlands
[13]Department of Vascular Surgery, Erasmus University Medical Centre, Rotterdam, The Netherlands
[14]Department of Surgery, Elisabeth-TweeSteden Hospital (ETZ), Tilburg, the Netherlands
[15] Department of Surgery, University Medical Centre Groningen, Groningen, the Netherlands
[16] Department of Surgery, Haga Hospital, The Hague, the Netherlands

For peer review only

CONSORT 2010 checklist of information to include when reporting a randomised trial*

Section/Topic	Item No	Checklist item	Reported on page No
**Title and abstract**			
	1a	Identification as a randomised trial in the title	n.a.
	1b	Structured summary of trial design, methods, results, and conclusions (for specific guidance see CONSORT for abstracts)	2
**Introduction**			
Background and objectives	2a	Scientific background and explanation of rationale	4,5,6
	2b	Specific objectives or hypotheses	7
**Methods**			
Trial design	3a	Description of trial design (such as parallel, factorial) including allocation ratio	8
	3b	Important changes to methods after trial commencement (such as eligibility criteria), with reasons	8,9
Participants	4a	Eligibility criteria for participants	9
	4b	Settings and locations where the data were collected	10
Interventions	5	The interventions for each group with sufficient details to allow replication, including how and when they were actually administered	n.a.
Outcomes	6a	Completely defined pre-specified primary and secondary outcome measures, including how and when they were assessed	10
	6b	Any changes to trial outcomes after the trial commenced, with reasons	n.a.
Sample size	7a	How sample size was determined	11
	7b	When applicable, explanation of any interim analyses and stopping guidelines	n.a.
Randomisation:			
Sequence generation	8a	Method used to generate the random allocation sequence	n.a.
	8b	Type of randomisation; details of any restriction (such as blocking and block size)	n.a.
Allocation concealment mechanism	9	Mechanism used to implement the random allocation sequence (such as sequentially numbered containers), describing any steps taken to conceal the sequence until interventions were assigned	n.a.
Implementation	10	Who generated the random allocation sequence, who enrolled participants, and who assigned participants to interventions	n.a.
Blinding	11a	If done, who was blinded after assignment to interventions (for example, participants, care providers, those	n.a.

For peer review only - http://bmjopen.bmj.com/site/about/guidelines.xhtml

		assessing outcomes) and how	
	11b	If relevant, description of the similarity of interventions	n.a.
Statistical methods	12a	Statistical methods used to compare groups for primary and secondary outcomes	12
	12b	Methods for additional analyses, such as subgroup analyses and adjusted analyses	12
**Results**			
Participant flow (a diagram is strongly recommended)	13a	For each group, the numbers of participants who were randomly assigned, received intended treatment, and were analysed for the primary outcome	n.a.
	13b	For each group, losses and exclusions after randomisation, together with reasons	n.a.
Recruitment	14a	Dates defining the periods of recruitment and follow-up	n.a.
	14b	Why the trial ended or was stopped	n.a.
Baseline data	15	A table showing baseline demographic and clinical characteristics for each group	n.a.
Numbers analysed	16	For each group, number of participants (denominator) included in each analysis and whether the analysis was by original assigned groups	n.a.
Outcomes and estimation	17a	For each primary and secondary outcome, results for each group, and the estimated effect size and its precision (such as 95% confidence interval)	n.a.
	17b	For binary outcomes, presentation of both absolute and relative effect sizes is recommended	n.a.
Ancillary analyses	18	Results of any other analyses performed, including subgroup analyses and adjusted analyses, distinguishing pre-specified from exploratory	n.a.
Harms	19	All important harms or unintended effects in each group (for specific guidance see CONSORT for harms)	n.a.
**Discussion**			
Limitations	20	Trial limitations, addressing sources of potential bias, imprecision, and, if relevant, multiplicity of analyses	13,14
Generalisability	21	Generalisability (external validity, applicability) of the trial findings	n.a.
Interpretation	22	Interpretation consistent with results, balancing benefits and harms, and considering other relevant evidence	n.a.
**Other information**			
Registration	23	Registration number and name of trial registry	2
Protocol	24	Where the full trial protocol can be accessed, if available	n.a.
Funding	25	Sources of funding and other support (such as supply of drugs), role of funders	15

*We strongly recommend reading this statement in conjunction with the CONSORT 2010 Explanation and Elaboration for important clarifications on all the items. If relevant, we also recommend reading CONSORT extensions for cluster randomised trials, non-inferiority and equivalence trials, non-pharmacological treatments, herbal interventions, and pragmatic trials. Additional extensions are forthcoming: for those and for up to date references relevant to this checklist, see www.consort-statement.org.

For peer review only - http://bmjopen.bmj.com/site/about/guidelines.xhtml

BMJ Open

Postoperative surveillance and long-term outcome after endovascular aortic aneurysm repair in the Netherlands: study protocol for the retrospective ODYSSEUS study.

Journal:	*BMJ Open*
Manuscript ID	bmjopen-2019-033584.R1
Article Type:	Protocol
Date Submitted by the Author:	26-Nov-2019
Complete List of Authors:	Geraedts, Anna; Amsterdam UMC - Locatie AMC, Surgery<br>de Mik, Sylvana; Amsterdam University Medical Centres, Surgery<br>Ubbink, Dirk; Amsterdam University Medical Centres, Surgery<br>Koelemay, Mark; Amsterdam University Medical Centres, Surgery<br>Balm, Ron; Amsterdam University Medical Centres, Surgery
**Primary Subject Heading**:	Surgery
Secondary Subject Heading:	Cardiovascular medicine
Keywords:	VASCULAR SURGERY, Aortic Aneurysm, Abdominal, Endovascular Procedures

SCHOLARONE™
Manuscripts

I, the Submitting Author has the right to grant and does grant on behalf of all authors of the Work (as defined in the below author licence), an exclusive licence and/or a non-exclusive licence for contributions from authors who are: i) UK Crown employees; ii) where BMJ has agreed a CC-BY licence shall apply, and/or iii) in accordance with the terms applicable for US Federal Government officers or employees acting as part of their official duties; on a worldwide, perpetual, irrevocable, royalty-free basis to BMJ Publishing Group Ltd ("BMJ") its licensees and where the relevant Journal is co-owned by BMJ to the co-owners of the Journal, to publish the Work in this journal and any other BMJ products and to exploit all rights, as set out in our licence.

The Submitting Author accepts and understands that any supply made under these terms is made by BMJ to the Submitting Author unless you are acting as an employee on behalf of your employer or a postgraduate student of an affiliated institution which is paying any applicable article publishing charge ("APC") for Open Access articles. Where the Submitting Author wishes to make the Work available on an Open Access basis (and intends to pay the relevant APC), the terms of reuse of such Open Access shall be governed by a Creative Commons licence – details of these licences and which Creative Commons licence will apply to this Work are set out in our licence referred to above.

Other than as permitted in any relevant BMJ Author's Self Archiving Policies, I confirm this Work has not been accepted for publication elsewhere, is not being considered for publication elsewhere and does not duplicate material already published. I confirm all authors consent to publication of this Work and authorise the granting of this licence.

For peer review only

Postoperative surveillance and long-term outcome after endovascular aortic aneurysm repair in the Netherlands: study protocol for the retrospective ODYSSEUS study.

A.C.M. Geraedts[1], S.M.L. de Mik[1], D.T. Ubbink[1], M.W.J. Koelemay[1], R. Balm[1], on behalf of the ODYSSEUS study group[2].

[1] Amsterdam University Medical Centres, University of Amsterdam, Amsterdam Cardiovascular Sciences, Department of Surgery, Meibergdreef 9, 1105AZ Amsterdam, The Netherlands

[2] Collaborator study group : ODYSSEUS study group.

COLLABORATORS TO THE ODYSSEUS STUDY GROUP (in alphabetical order):

J.W. Elshof[1], B.H.P. Elsman[2], J.F. Hamming[3], J.A. van Herwaarden[4], R.H.J. Kropman[5], M.M. Lensvelt[6], P.P. Poyck[7], G.W.H. Schurink[8], A.A.E.A de Smet[9], S.M. van Sterkenburg[10], C. Ünlü[11], A.C. Vahl[12], H.J.M. Verhagen[13], P.W.H.E. Vriens[14], J.P.P.M. de Vries[5,15], J.J. Wever[16], W. Wisselink[6], C.J. Zeebregts[15]

[1]Department of Surgery, VieCuri Medical Centre, Venlo, the Netherlands
[2]Department of Surgery, Deventer Hospital, Deventer, the Netherlands
[3]Department of Surgery, Leiden University Medical Centre, Leiden, the Netherlands
[4]Department of Surgery, University Medical Centre Utrecht, Utrecht, the Netherlands
[5]Department of Surgery, St. Antonius Hospital, Nieuwegein, the Netherlands
[6]Department of Surgery, Amsterdam University Medical Centres, Amsterdam Cardiovascular Sciences, Amsterdam, the Netherlands
[7]Department of Vascular and Transplant Surgery, Radboud University Medical Centre, Nijmegen, the Netherlands
[8]Department of Vascular Surgery, Maastricht University Medical Centre, Maastricht, the Netherlands
[9]Department of Surgery, Maasstad Hospital, Rotterdam, the Netherlands
[10]Department of Surgery, Rijnstate Hospital, Arnhem, the Netherlands
[11]Department of Surgery, Noordwest Ziekenhuisgroep, Alkmaar, the Netherlands
[12]Department of Surgery, OLVG, Amsterdam, The Netherlands
[13]Department of Vascular Surgery, Erasmus University Medical Centre, Rotterdam, the Netherlands
[14]Department of Surgery, Elisabeth-TweeSteden Hospital (ETZ), Tilburg, the Netherlands

For peer review only

[15]Department of Surgery, University Medical Centre Groningen, Groningen, the Netherlands
[16]Department of Surgery, Haga Hospital, The Hague, the Netherlands

Contact details for corresponding author and reprints:

A.C.M. Geraedts, MD

Address: Amsterdam University Medical Centres, University of Amsterdam, Amsterdam Cardiovascular Sciences, Department of Surgery, Meibergdreef 9, 1105AZ Amsterdam, The Netherlands

Phone: +31(0)205662971

E-mail: a.c.geraedts@amsterdamumc.nl

Word count: 3421

Keywords:

Vascular Surgical Procedures; Aortic Aneurysm, Abdominal; Endovascular Procedures**.**

For peer review only

ABSTRACT

Introduction: Strict imaging surveillance protocols to detect complications following Endovascular Aneurysm Repair (EVAR) are common practice. However, controversy exists as to whether all EVAR patients need intense surveillance. The 2019 European Society for Vascular Surgery (ESVS) guidelines for management of abdominal aortic aneurysm (AAA) suggest that patients may be considered for limited follow-up with imaging if classified as 'low risk' for complications based on their initial postoperative imaging. The current study aims to investigate the intervention-free survival and overall survival stratified for patients with and without yearly imaging surveillance.

Methods and analysis: The ODYSSEUS study comprises a national multicentre retrospective cohort study in 17 medical centres. Consecutive patients with an asymptomatic or symptomatic infrarenal AAA who underwent EVAR between January 2007 and January 2012 will be included in this study with follow-up until December 2018. Clinical variables and all follow-up information will be retrieved in extensive data collection from the patient's medical records. In addition, an e-survey was sent to vascular surgeons at the 17 participating centres to gauge their opinions regarding the possibility of safely reducing the frequency of imaging surveillance. Primary endpoints are intervention after EVAR and aneurysm related mortality. The initial estimated sample size is 1997 patients.

Ethics and dissemination: The study has been approved by the Medical Ethics Review Committee of the Amsterdam UMC, location Academic Medical Centre, Amsterdam, the Netherlands. Study findings will be disseminated via presentations at conferences and publications in peer-reviewed journal.

Registration: Netherlands Trial Registry, NL6953 (old: NTR28773). Registered 5 Apr 2018. URL: https://www.trialregister.nl/trial/6953

For peer review only

ARTICLE SUMMARY

Strengths and limitations of this study

- The main strength of this study is that it can accumulate data from large number of patients with long-term follow-up up to 11 years and that it captures all surveillance visits, long-term outcomes and mortality post-EVAR.
- The sample size will be large enough to enable survival and regression analyses in small sub-groups of patients.
- The main limitation of the study is due to the nature of retrospective data, it allows only the collection of data that was documented in the patient medical records.

For peer review only

INTRODUCTION

Endovascular Aneurysm Repair (EVAR) of the abdominal aorta has become the primary treatment of patients with an abdominal aortic aneurysm (AAA).[1] Both the Society for Vascular Surgery (SVS) International Guidelines and the instructions for use (IFU) of endograft manufacturers recommend yearly imaging surveillance for all patients after EVAR.[2] However, if the patient is classified as 'low risk' for complications based on initial post-operative imaging, the 2019 European Society for Vascular Surgery (ESVS) guidelines recommend delaying imaging until five years after repair.[3] This movement towards reducing the imaging frequency will benefit patients, medical centres and health care costs.

Imaging surveillance by Computed Tomography Angiography (CTA) may increase the attributable lifetime cancer risk of patients, as well as putting them at risk of developing nephropathy due to contrast exposure. If yearly CTA is replaced by duplex ultrasonography (DUS) patients still experience the burden of additional hospital visits. Moreover, compliance with yearly imaging is suboptimal and non-adherence to yearly imaging does not appear to be associated with poorer outcomes.[4][5]

It has been questioned whether yearly imaging is necessary for all EVAR patients, and if a specific group of patients can be identified for which surveillance intervals can safely be extended, as is suggested by the new guideline.[3] For these reasons, in the Netherlands the Observing a Decade of Yearly Standardised Surveillance in EVAR patients with Ultrasound or CT Scan (ODYSSEUS) study has been designed. In this study of approximately 2000 patients with 6-11 years of follow-up, we aim to determine when, and in which patients, it is safe to deviate from the current annual surveillance protocols.

For peer review only

Background and relevant literature and data

Before initiating the ODYSSEUS study, we conducted a survey among Dutch vascular surgeons to find out if they support the possibility of reducing the frequency of imaging surveillance. In this survey, vascular surgeons reported the main reasons patients did not comply with follow-up visits, i.e. they had forgotten the appointment or were prevented by force majeure. Most physicians estimated that less than 10% of their patients had missed one or more follow-up visits post-EVAR. This might be an overestimation of the true adherence to follow-up visits, as these observations are in contrast with a study reporting that only 43% of patients had complete surveillance.[4]

We also asked participating vascular surgeons to upload their standard post-EVAR protocol to investigate if there were differences between centres in the Netherlands. In all centres imaging took place within the first 3 months after surgery, mostly by CTA. Most centres comply with their own post-EVAR surveillance protocols, which have many commonalities with the SVS and ESVS guidelines. Only one centre utilizes precisely the same post-EVAR surveillance protocol as recommended by the SVS guidelines. Another centre had already reduced follow-up imaging to once every five years, using either CTA or DUS as is stated in the new ESVS guidelines.[3] While vascular surgeons still seem to adhere to their hospital-specific protocol, they do support the need for reducing follow-up by selecting a group of patients for which yearly follow-up can safely be omitted. However, some surgeons indicated that more evidence is needed than is available in the current literature.[6][7]

In studies that have investigated the indications for post-EVAR intervention, it is stated that 61-98% of interventions were necessary because of symptoms and not because of findings at surveillance imaging. This suggests that post-EVAR surveillance protocols provide no benefit to a large group of patients, as complications occur in between surveillance visits.[8][9] Imaging surveillance may even lead to unnecessary interventions and it does not appear to be associated with improved survival.[9][10] We hypothesize that the requirement

For peer review only

for routine imaging for patients at low risk can be reduced. However, novel endovascular devices still require more intensive surveillance as the short- and long-term results of those devices remain undetermined.

For peer review only

STUDY OBJECTIVES

The objective of this study is to evaluate whether imaging surveillance frequency can be safely reduced in a selected group of EVAR patients, for example in patients with an asymptomatic or symptomatic infrarenal AAA who underwent EVAR and who had no abnormalities on the first postoperative CTA. The clinical course of a large cohort of patients will be evaluated with follow-up ranging between 6 and 11 years. Baseline patient characteristics, aortic anatomy and details of the operation will be derived from the patient's medical record. The first milestone during follow-up is the first postoperative CTA. This scan either shows complications such as endoleaks, malposition or migration of the graft, or the absence thereof. All follow-up visits, imaging studies, as well as all interventions after EVAR and outcomes will be registered. Our hypothesis is that patients with less follow-up will have better outcomes regarding the number of interventions and aneurysm related mortality compared to patients with annual follow-up. Regarding the intervention rates, it is expected that adherence to imaging surveillance may detect more abnormalities triggering re-interventions, which in itself may cause additional complications and perhaps even a decrease in survival rates. We hypothesize that the need for routine imaging for patients with no abnormalities at their initial CTA can be decreased.

For peer review only

METHODS AND ANALYSIS

The study protocol has been designed according to the Standard Protocol Items: Recommendations for Interventional Trials (SPIRIT) statement and the CONsolidated Standards of Reporting Trials (CONSORT).[11,12] The study is registered in The Netherlands National Trial Registry as registration number NL6953**,** available at: https://www.trialregister.nl/trial/6953

General study design

A multicentre retrospective cohort study in 17 medical centres in the Netherlands. Data will be collected from the medical records of all consecutive patients with AAA who underwent EVAR between January 2007 and January 2012. This selection provides a theoretical length of follow-up of 6-11 years on December 2018. Patients will be divided into three groups: A) patients without abnormalities at their first postoperative CTA with yearly imaging surveillance, B) patients without abnormalities at their first postoperative CTA without yearly imaging surveillance and C) patients with abnormalities at their first postoperative CTA (Figure 1). This retrospective design has the advantage of collecting long term follow-up data. The Dutch Dream trial found that the number of interventions starts to rise 4 years after EVAR and the long-term results of the EVAR-1 trial show that EVAR has an early survival benefit but inferior late survival compared with open surgical repair.[13,14] This is in contrast to the recently published long-term results of the OVER trial in which no difference was observed between EVAR and OSR in the primary outcome of all-cause mortality.[15] Hence, a prospective study would take approximately 8 to 10 years to gather enough patients with adequate follow-up.

The standard of care is defined by the current guidelines and instructions for use (IFU). The usual follow-up schedule in the IFU is: CTA and abdominal X-ray at 30 days, 6 and 12 months and yearly thereafter. The 2019 ESVS guidelines recommend a CTA 30 days after

For peer review only

EVAR. If there is adequate seal and no endoleak patients are classified as low risk and CTA follow-up may take place 5 years later. If there is an inadequate seal and endoleak type I/III patients could be either evaluated for re-intervention or if sac shrinkage occurs yearly DUS is recommended. In the 2018 SVS guideline CTA at one and 12 months is recommended and if neither endoleak nor sac enlargement is documented, DUS is suggested for annual postoperative surveillance. In our study design the definition of compliance is undergoing imaging surveillance every 16 months since patients in most centres will be rescheduled if they missed their annual follow-up visit. Device-specific complications after EVAR will also be examined.

Study population

Patients eligible for this retrospective study are all adults who underwent elective EVAR for asymptomatic or symptomatic infrarenal AAA between January 2007 and January 2012. Table 1 gives a more detailed overview of the inclusion and exclusion criteria.

Patient and public involvement

No patients were involved in the research design and conception of this research study.

Date range of the study

Data will be extracted from patient medical records retrospectively and entered into a database with data validation from December 2018 until June 2020. At first two researchers will extract data together to standardize data extraction. Next, to further improve the validity of the data two researchers will independently extract data and enter it into the secured data base. Disagreements will be noted and resolved by discussion and if necessary by asking another co-author to act as an arbiter.

Subject selection

For peer review only

A retrospective cohort study of consecutive patients treated at 17 vascular centres is to be performed. All patients are eligible and the opt-out procedure will be used to allow patients to object to participation within four weeks, which is in accordance with the Dutch Code of Civil Procedure. The Medical Ethics Review Committee of the Amsterdam UMC, location Academic Medical Centre, Amsterdam, has confirmed that the Medical Research Involving Human Subjects Act (WMO) does not apply to our study. This study is conducted according to the General Data Protection Regulation (AVG 2016) and the Medical Treatment Agreement Act (WGBO).

Data sources

Paper or electronic medical records are used in order to identify participants who match study-defined criteria.

Primary and secondary endpoints

Main study endpoint:

- The number of patients with an intervention and aneurysm related mortality classified for patients with and without yearly imaging surveillance.

Secondary study endpoints:

- Date, type, indication and outcome of all postoperative imaging during follow-up.
- Type I, type II, type II and type IV endoleak, graft or outflow (iliac) occlusion, endograft infection detected by postoperative imaging, if present.
- Date and type of intervention during follow-up, if present.
- Date of aneurysm rupture during follow-up, if present.
- Date of death during follow-up, if present.
- Costs of all EVAR related imaging and outpatient clinic visits.

For peer review only

Study procedures

The primary outcomes of this study are interventions and aneurysm related mortality for patients who had a normal initial postoperative CTA and who do adhere to our definition of yearly imaging surveillance over a 6 to 11-year follow-up period, compared to those who do not adhere to our definition.

Interventions are EVAR-related interventions defined by the SVS reporting standards as postoperative adjunctive manoeuvres.[16][17] Interventions for wound complications at the access site are not included, since these are detectable without the use of imaging.

Date of death during follow-up, if applicable, will be obtained from patient medical records and verified by the Dutch municipal personal records database (GBA).

Details of all surveillance imaging are obtained from patient medical records and radiology reports. The time between imaging appointments is calculated to determine whether patients adhere to this studies definition of with yearly imaging surveillance, i.e. within every 16 months.

Date, type, indication and outcome of all postoperative imaging during follow-up are obtained from patient medical records, specifically imaging order forms and radiology reports. A normal initial postoperative CT scan is defined as a CT scan which shows no endoleaks, endograft migration (>10 mm), kinking or obstruction. All imaging outcomes are based on the report compiled by radiologists. These reports will not be re-evaluated by an independent radiologist, since we want to base our outcomes on real life data.

Secondary outcomes are all-cause mortality, type I, type II, type III and type IV endoleak, graft or outflow (iliac) occlusion, aneurysm rupture and endograft infection. This is also obtained from patient medical records, specifically radiology reports and Dutch municipal personal records database (GBA).

Date of aneurysm rupture is obtained from patient medical records, specifically operative reports, radiology reports and progress notes.

For peer review only

Costs of all EVAR-related imaging and outpatient clinic visits will be calculated per patient. Cost is defined as volume times price. Prices from the "Cost manual of the Dutch Health Care Institute" will be used. Costs for the patients will also be included. The quality-adjusted life years (QALYs), a generic measure of disease burden including both the quality and quantity of life, cannot be calculated with this retrospective design.

Sample size and power

Sample size calculation for this study is based on an expected difference of 7% in the proportions of patients not requiring interventions after 7 years between patients undergoing yearly standardised imaging surveillance (75% intervention-free rate [18]) versus those s not undergoing standardised imaging surveillance(82% intervention-free rate[19]). To detect this difference with 90% power and a 0.05 significance level, 719 patients per group are required and 1438 in total. To correct for the fact that the first CTA of approximately 20% of patients is abnormal, 1798 patients (1438/0.8) are needed.[20][21] In addition, we expect incomplete data in 10% of the patients which results in a total number of 1997 patients(1798/0.9).. With this sample size we can also detect a 3% difference in aneurysm related mortality with statistical significance. We chose a one-sided significance level (non-inferiority) of 0.05 and for standard proportion a 95% non-aneurysm related mortality and thus 5% freedom from aneurysm-related mortality after 7 years: 0.95. For equivalence limit difference, we chose an acceptable difference between groups of 3%, in which if differences in aneurysm related mortality equals 3%, they are considered non-inferior. Test-expected proportion is then equal to the standard proportion 0.97. Thus, the expected difference is 0, calculated with a power of 80%. This results in 653 patients per group and 1306 patients in total. Since the first CTA of approximately 20% of patients is abnormal, 1632 patients (1306/0.8) are needed. In addition, we expect incomplete data in

For peer review only

10% of the patients which results in a minimum of, 1813 patients (1632/0.9) are needed in total.

Statistical methods

Differences in baseline characteristics between patients undergoing or not undergoing yearly standardised imaging surveillance by either CTA or DUS, will be analysed using the Chi-square or Fisher's exact test for categorical variables and the student's t-test or Mann Whitney test for continuous variables, if appropriate.

The primary endpoints, i.e. survival and freedom from intervention will be estimated by Kaplan-Meier survival analysis and differences between groups will be assessed with the log-rank test.

Secondary endpoints such as freedom from aneurysm rupture between patients with and without yearly standardised imaging surveillance will be estimated by Kaplan-Meier survival analysis, and differences between these groups will be calculated with the log-rank test.

Multivariable cox regression analysis will be used to determine survival and the freedom of intervention corrected for age, gender, AAA diameter, ASA classification, neck length, neck angulation and type of endograft. The association between postoperative intervention and the following covariates will be investigated with the multivariate Cox-regression analysis:

- age
- gender
- AAA diameter
- ASA classification
- neck length (>15mm)
- neck angulation (>60°)

For peer review only

- type of endograft
- initial postoperative CTA

All statistical analyses will be done with SPSS software (IBM, version 25). The level for statistical significance is set at a p-value < 0.05.

For peer review only

DISCUSSION

The goal of this study is to evaluate whether a reduction in follow-up visits and imaging and thus costs, in patients operated on for an asymptomatic or symptomatic AAA with EVAR is safe. We hypothesize that there will be less interventions and no difference in aneurysm related mortality in patients with less intensive follow-up. With the results of this study we aim to provide scientific evidence helping vascular surgeons decide whether less vigilant follow-up after EVAR may be considered for patients classified in the low risk group.

The strengths of the ODYSSEUS study are that it can accumulate the data of a large number of consecutive EVAR patients with a theoretical follow-up of 6 to 11 years, and that it captures all surveillance visits, long-term outcomes and mortality post-EVAR. Moreover, 17 medical centres throughout the Netherlands are participating in this study, including university and general hospitals, thereby reducing selection bias.

An e-survey has been sent to all vascular surgeons participating in the ODYSSEUS study. This shows that yearly imaging surveillance is upheld by most vascular surgeons in the Netherlands. In addition, most physicians agree that yearly imaging frequency can be safely reduced in a specific group of EVAR patients. As support for this reduction in frequency is evident in the Netherlands, the next step will be to study the groups for which it will be safe to deviate from the widely accepted surveillance protocols.

Our study is subject to limitations due to the nature of administrative data and its retrospective and observational design. As with all studies using administrative data, it allows only the collection of data that was documented in patient medical records. It is also possible that some patients may have transferred to alternative surveillance protocols in different medical centres without our knowledge. The study will assess results in 17 medical centres over 11 years, during which time improvements in endograft and in clinical practice has occurred. Attrition bias due to loss to follow up represents a threat to the internal validity of our cohort study. The mentioned e-survey has only been sent to participating vascular

For peer review only

surgeons, perhaps surgeons participating in the ODYSSEUS study strongly believe that imaging surveillance frequency can be reduced. This may have provided a biased view of post-EVAR follow-up in the Netherlands. However, most of the high-volume EVAR centres in the Netherlands have been included.

In conclusion, with the ODYSSEUS study we aim to confirm the follow-up protocol of the recent ESVS guideline delaying imaging after 5 years if classified in the low risk group and therefore aim to investigate the intervention-free-survival and aneurysm related mortality for patients with and without yearly imaging surveillance.

For peer review only

STUDY ADMINISTRATION

Ethics

The Medical Ethics Review Committee of the Academic Medical Centre, Amsterdam, has reviewed and approved our study protocol version 1.6 dated 26 March 2018. The study is being conducted according to the principles of the Declaration of Helsinki in the current version of Fortaleza, Brazil (2013). Principles of good clinical practice will be respected. Study participation is voluntary.

Data management

All data obtained during the course of the study are considered to be confidential and will not be distributed to third parties. Patient data are stored anonymously under a code. Only the principal investigator or researchers authorised by the principal investigator have access to the key file.

Informed consent

For this retrospective study no informed consent is required. Patients are informed by the researchers that their patient information will be used for research. Patients are able to opt-out of their information being used by returning the opt-out form attached to the patient information letter via email or stamped return envelopes.

Funding statement

This study is funded by the AMC Foundation and with financial support of the Netherlands Organization for Health Research and Development (ZonMw; grant 843004119). The duration of the study is from July 15, 2018 to July 15, 2021. The AMC Foundation nor ZonMw is involved in the study design, writing of the manuscript or the decision to submit the manuscript for publication.

For peer review only

Conflict of interest

The authors declare that they have no competing interests.

Dissemination

We aim to produce high-impact peer-reviewed publications of the results of the study and present our findings at national and international conferences. The members of the project group of this study will be involved in preparing manuscript drafts and abstract among any other publications arising from the study. The Netherlands Organization for Health Research and Development demands us to stay in close cooperation with the patients association ('Harteraad'). The results of this study will be shared with the members of the patients association via multiple modalities.

Author contributions

Conception and design of study: A.C.M. Geraedts, S.M.L. de Mik, D.T. Ubbink, M.W.J. Koelemay, R. Balm.

Drafting the manuscript: A.C.M. Geraedts, S.M.L. de Mik.

Revising the manuscript critically for important intellectual content: D.T. Ubbink, M.W.J. Koelemay, R. Balm.

Approval of the version of the manuscript to be published: A.C.M. Geraedts, S.M.L. de Mik, D.T. Ubbink, M.W.J. Koelemay, R. Balm.

Agreement to be accountable for all aspects of the work: A.C.M. Geraedts, S.M.L. de Mik, D.T. Ubbink, M.W.J. Koelemay, R. Balm.

Data statement

Castor EDC, the Netherlands will be used for data collection and will be managed by qualified personnel. The data output can be opened in SPSS. Data is published via an DOI-code that

For peer review only

will be requested via Figshare. Storage of the data under the DOI-code is one of the requirements of ZonMw.

For peer review only

REFERENCES

1.The EVAR trial participants. Comparison of endo vascular aneurysm repair with open repair in patients with abdominal aortic aneurysm (EVAR trial 1), 30-day operative mortality results: randomized controlled trial. Lancet 2004; 364: 843–48.

2. Chaikof EL, Brewster DC, Dalman RL, et al. SVS Practice guidelines for the care of patients with an abdominal aortic aneurysm: executive summary. J Vasc Surg 2018;67(1):2-77..

3. Wanhainen A, Verzini F, van Herzeele I, et al. European Society for Vascular Surgery (ESVS) 2019 Clinical Practice Guidelines on the Management of Abdominal Aorto-Iliac Artery Aneurysms. Eur J Vasc Endovasc Surg. 2019 Jan;57(1):8-93.

4. Garg T, Baker LC, Mell MW. Postoperative Surveillance and long-term outcomes after endovascular aneurysm repair among Medicare beneficiaries. JAMA Surg 2015;150:957-63.

5. Kret MR, Azarbal AF, Mitchell EL, et al. Compliance with long-term surveillance recommendations following endovascular aneurysm repair or type B aortic dissection. J Vasc Surg 2013 Jul;58(1):25-31.

6. Baderkhan H, Haller O, Wanhainen A, Björck M, Mani K. Follow-up after endovascular aortic aneurysm repair can be stratified based on first postoperative imaging. Br J Surg 2018;105:709-18

7. Bastos Goncalves F, Baderkhan H, Verhagen HJ, Wanhainen A, Björck M, Stolker RJ, et al. Early sac shrinkage predicts a low risk of late complications after endovascular aortic aneurysm repair. Br J Surg 2014;101:802-10.

8. Karthikesalingam A, Holt PJ, Hinchliffe RJ, et al. Risk of reintervention after endovascular aortic aneurysm repair. Br J Surg 2010;97(5):657-63.

9. Dias NV, Riva L, Ivancev K, et al. Is there a benefit of frequent CT follow-up after EVAR? Eur J Vasc Endovasc Surg 2009;37(4):425-30.

10. De Mik SML, Geraedts ACM, Ubbink DT, et al. Effect of imaging surveillance after Endovascular Aneurysm Repair on reinterventions and mortality: a systematic review and meta-analysis. J Endovasc Ther. 2019;26(4):531-541.

For peer review only

11.Chan AW, Tetzlaff JM, Gotzsche PC, Altman DG, Mann H, Berlin JA, et al. SPIRIT 2013 explanation and elaboration: guidance for protocols of clinical trials. BMJ. 2013;346.

12. Campbell MK, Piaggio G, Elbourne DR, Altman DG, Group C. Consort 2010 statement: extension to cluster randomised trials. BMJ. 2012;345.

13. De Bruin Jorg L, Baas Annette F, Buth Jaap, et al, for the DREAM Study Group. Long-term outcome of open or endovascular repair of abdominal aortic aneurysm. N Engl J Med 2010;362:1881e9.

14. Patel R, Powell JT, Sweeting MJ, et al. The UK EndoVascular Aneurysm Repair (EVAR) randomised controlled trials: long-term follow-up and cost-effectiveness analysis. Health Technol Assess. 2018 Jan;22(5):1-132.

15. Lederle FA, Kyriakides TC, Stroupe, KT, et al. Open versus endovascular repair of abdominal aortic aneurysm. N Engl J Med 2019;380(22):2126-2135.

16. Chaikof EL, Blankensteijn JD, Harris PL, et al. Reporting standards for endovascular aortic aneurysm repair. J Vasc Surg. 2002;35(5):1048-60.

17. Alonso J, Bartlett SJ, Rose M, et al. The case for an international patient-reported outcome measurement information system (PROMIS®) initiative. Health Qual life Outcomes 11:210, 2013.

18. Wu CY, Chen H, Gallagher KA, et al. Predictors of compliance with surveillance after endovascular aneurysm repair and comparative survival outcomes. J Vasc Surg. 2015;62(1):27-35.

19. Conrad MF, Adams AB, Guest JM, et al. Secondary intervention after endovascular abdominal aortic aneurysm repair. Ann Surg. 2009;250(3):383-9.

20. Patel MS, Carpenter JP. The value of the initial post-EVAR computed tomography angiography scan in predicting future secondary procedures using the Powerlink stent graft. J Vasc Surg. 2010;52(5):1135-9.

For peer review only

21. Troutman DA, Chaudry M, Dougherty MJ, et al. Endovascular aortic aneurysm repair surveillance may not be necessary for the first 3 years after an initially normal duplex postoperative study. J Vasc Surg. 2014;60(3):558-62.

For peer review only

Tables

Table 1

Retrospective cohort

Inclusion criteria	Exclusion criteria
Age ≥ 18 years<br>Patient with an (a)asymptomatic infrarenal abdominal aortic aneurysm<br>EVAR between January 2007 and January 2012<br>Patients with an initial postoperative CTA within 90 days after EVAR	Connective tissue disease<br>Patients who objected to their retrospective data being used

For peer review only

Figures

Figure 1. Patient subgroups

For peer review only

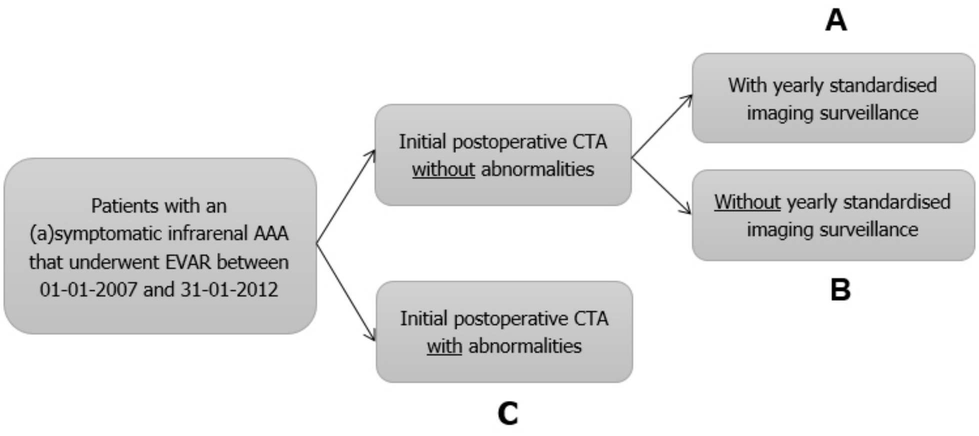

Figure 1. Patient subgroups

STROBE Statement—checklist of items that should be included in reports of observational studies

	Item No	Recommendation	Page No
**Title and abstract**	1	(*a*) Indicate the study's design with a commonly used term in the title or the abstract	1
		(*b*) Provide in the abstract an informative and balanced summary of what was done and what was found	3
**Introduction**			
Background/rationale	2	Explain the scientific background and rationale for the investigation being reported	4
Objectives	3	State specific objectives, including any prespecified hypotheses	7
**Methods**			
Study design	4	Present key elements of study design early in the paper	9
Setting	5	Describe the setting, locations, and relevant dates, including periods of recruitment, exposure, follow-up, and data collection	10-12
Participants	6	(*a*) *Cohort study*—Give the eligibility criteria, and the sources and methods of selection of participants. Describe methods of follow-up *Case-control study*—Give the eligibility criteria, and the sources and methods of case ascertainment and control selection. Give the rationale for the choice of cases and controls *Cross-sectional study*—Give the eligibility criteria, and the sources and methods of selection of participants	9,10
		(*b*) *Cohort study*—For matched studies, give matching criteria and number of exposed and unexposed *Case-control study*—For matched studies, give matching criteria and the number of controls per case	n.a.
Variables	7	Clearly define all outcomes, exposures, predictors, potential confounders, and effect modifiers. Give diagnostic criteria, if applicable	11,12
Data sources/ measurement	8*	For each variable of interest, give sources of data and details of methods of assessment (measurement). Describe comparability of assessment methods if there is more than one group	12,13
Bias	9	Describe any efforts to address potential sources of bias	16,17
Study size	10	Explain how the study size was arrived at	13
Quantitative variables	11	Explain how quantitative variables were handled in the analyses. If applicable, describe which groupings were chosen and why	14
Statistical methods	12	(*a*) Describe all statistical methods, including those used to control for confounding	14
		(*b*) Describe any methods used to examine subgroups and interactions	14
		(*c*) Explain how missing data were addressed	13,14
		(*d*) *Cohort study*—If applicable, explain how loss to follow-up was addressed *Case-control study*—If applicable, explain how matching of cases and controls was addressed *Cross-sectional study*—If applicable, describe analytical methods taking account of sampling strategy	16
		(*e*) Describe any sensitivity analyses	n.a.

Continued on next page

For peer review only - http://bmjopen.bmj.com/site/about/guidelines.xhtml

**Results**			
Participants	13*	(a) Report numbers of individuals at each stage of study—eg numbers potentially eligible, examined for eligibility, confirmed eligible, included in the study, completing follow-up, and analysed	N.a.
		(b) Give reasons for non-participation at each stage	N.a.
		(c) Consider use of a flow diagram	N.a.
Descriptive data	14*	(a) Give characteristics of study participants (eg demographic, clinical, social) and information on exposures and potential confounders	N.a.
		(b) Indicate number of participants with missing data for each variable of interest	N.a.
		(c) *Cohort study*—Summarise follow-up time (eg, average and total amount)	N.a.
Outcome data	15*	*Cohort study*—Report numbers of outcome events or summary measures over time	N.a.
		Case-control study—Report numbers in each exposure category, or summary measures of exposure	N.a.
		Cross-sectional study—Report numbers of outcome events or summary measures	N.a.
Main results	16	(*a*) Give unadjusted estimates and, if applicable, confounder-adjusted estimates and their precision (eg, 95% confidence interval). Make clear which confounders were adjusted for and why they were included	N.a.
		(*b*) Report category boundaries when continuous variables were categorized	N.a.
		(*c*) If relevant, consider translating estimates of relative risk into absolute risk for a meaningful time period	N.a.
Other analyses	17	Report other analyses done—eg analyses of subgroups and interactions, and sensitivity analyses	N.a.
**Discussion**			
Key results	18	Summarise key results with reference to study objectives	16
Limitations	19	Discuss limitations of the study, taking into account sources of potential bias or imprecision. Discuss both direction and magnitude of any potential bias	16
Interpretation	20	Give a cautious overall interpretation of results considering objectives, limitations, multiplicity of analyses, results from similar studies, and other relevant evidence	16,17
Generalisability	21	Discuss the generalisability (external validity) of the study results	16,17
**Other information**			
Funding	22	Give the source of funding and the role of the funders for the present study and, if applicable, for the original study on which the present article is based	18

*Give information separately for cases and controls in case-control studies and, if applicable, for exposed and unexposed groups in cohort and cross-sectional studies.

Note: An Explanation and Elaboration article discusses each checklist item and gives methodological background and published examples of transparent reporting. The STROBE checklist is best used in conjunction with this article (freely available on the Web sites of PLoS Medicine at http://www.plosmedicine.org/, Annals of Internal Medicine at http://www.annals.org/, and Epidemiology at http://www.epidem.com/). Information on the STROBE Initiative is available at www.strobe-statement.org.

For peer review only

BMJ Open

Postoperative surveillance and long-term outcome after endovascular aortic aneurysm repair in the Netherlands: study protocol for the retrospective ODYSSEUS study.

Journal:	*BMJ Open*
Manuscript ID	bmjopen-2019-033584.R2
Article Type:	Protocol
Date Submitted by the Author:	31-Dec-2019
Complete List of Authors:	Geraedts, Anna; Amsterdam UMC - Locatie AMC, Surgery<br>de Mik, Sylvana; Amsterdam University Medical Centres, Surgery<br>Ubbink, Dirk; Amsterdam University Medical Centres, Surgery<br>Koelemay, Mark; Amsterdam University Medical Centres, Surgery<br>Balm, Ron; Amsterdam University Medical Centres, Surgery
**Primary Subject Heading**:	Surgery
Secondary Subject Heading:	Cardiovascular medicine
Keywords:	VASCULAR SURGERY, Aortic Aneurysm, Abdominal, Endovascular Procedures

SCHOLARONE™
Manuscripts

I, the Submitting Author has the right to grant and does grant on behalf of all authors of the Work (as defined in the below author licence), an exclusive licence and/or a non-exclusive licence for contributions from authors who are: i) UK Crown employees; ii) where BMJ has agreed a CC-BY licence shall apply, and/or iii) in accordance with the terms applicable for US Federal Government officers or employees acting as part of their official duties; on a worldwide, perpetual, irrevocable, royalty-free basis to BMJ Publishing Group Ltd ("BMJ") its licensees and where the relevant Journal is co-owned by BMJ to the co-owners of the Journal, to publish the Work in this journal and any other BMJ products and to exploit all rights, as set out in our licence.

The Submitting Author accepts and understands that any supply made under these terms is made by BMJ to the Submitting Author unless you are acting as an employee on behalf of your employer or a postgraduate student of an affiliated institution which is paying any applicable article publishing charge ("APC") for Open Access articles. Where the Submitting Author wishes to make the Work available on an Open Access basis (and intends to pay the relevant APC), the terms of reuse of such Open Access shall be governed by a Creative Commons licence – details of these licences and which Creative Commons licence will apply to this Work are set out in our licence referred to above.

Other than as permitted in any relevant BMJ Author's Self Archiving Policies, I confirm this Work has not been accepted for publication elsewhere, is not being considered for publication elsewhere and does not duplicate material already published. I confirm all authors consent to publication of this Work and authorise the granting of this licence.

For peer review only

Postoperative surveillance and long-term outcome after endovascular aortic aneurysm repair in the Netherlands: study protocol for the retrospective ODYSSEUS study.

A.C.M. Geraedts[1], S.M.L. de Mik[1], D.T. Ubbink[1], M.W.J. Koelemay[1], R. Balm[1], on behalf of the ODYSSEUS study group[2].

[1] Amsterdam University Medical Centres, University of Amsterdam, Amsterdam Cardiovascular Sciences, Department of Surgery, Meibergdreef 9, 1105AZ Amsterdam, The Netherlands

[2] Collaborator study group : ODYSSEUS study group.

COLLABORATORS TO THE ODYSSEUS STUDY GROUP (in alphabetical order):

J.W. Elshof[1], B.H.P. Elsman[2], J.F. Hamming[3], J.A. van Herwaarden[4], R.H.J. Kropman[5], M.M. Lensvelt[6], P.P. Poyck[7], G.W.H. Schurink[8], A.A.E.A de Smet[9], S.M. van Sterkenburg[10], C. Ünlü[11], A.C. Vahl[12], H.J.M. Verhagen[13], P.W.H.E. Vriens[14], J.P.P.M. de Vries[5,15], J.J. Wever[16], W. Wisselink[6], C.J. Zeebregts[15]

[1]Department of Surgery, VieCuri Medical Centre, Venlo, the Netherlands
[2]Department of Surgery, Deventer Hospital, Deventer, the Netherlands
[3]Department of Surgery, Leiden University Medical Centre, Leiden, the Netherlands
[4]Department of Surgery, University Medical Centre Utrecht, Utrecht, the Netherlands
[5]Department of Surgery, St. Antonius Hospital, Nieuwegein, the Netherlands
[6]Department of Surgery, Amsterdam University Medical Centres, Amsterdam Cardiovascular Sciences, Amsterdam, the Netherlands
[7]Department of Vascular and Transplant Surgery, Radboud University Medical Centre, Nijmegen, the Netherlands
[8]Department of Vascular Surgery, Maastricht University Medical Centre, Maastricht, the Netherlands
[9]Department of Surgery, Maasstad Hospital, Rotterdam, the Netherlands
[10]Department of Surgery, Rijnstate Hospital, Arnhem, the Netherlands
[11]Department of Surgery, Noordwest Ziekenhuisgroep, Alkmaar, the Netherlands
[12]Department of Surgery, OLVG, Amsterdam, The Netherlands
[13]Department of Vascular Surgery, Erasmus University Medical Centre, Rotterdam, the Netherlands
[14]Department of Surgery, Elisabeth-TweeSteden Hospital (ETZ), Tilburg, the Netherlands

For peer review only

[15]Department of Surgery, University Medical Centre Groningen, Groningen, the Netherlands
[16]Department of Surgery, Haga Hospital, The Hague, the Netherlands

Contact details for corresponding author and reprints:

A.C.M. Geraedts, MD

Address: Amsterdam University Medical Centres, University of Amsterdam, Amsterdam Cardiovascular Sciences, Department of Surgery, Meibergdreef 9, 1105AZ Amsterdam, The Netherlands

Phone: +31(0)205662971

E-mail: a.c.geraedts@amsterdamumc.nl

Word count: 3505

Keywords:

Vascular Surgical Procedures; Aortic Aneurysm, Abdominal; Endovascular Procedures.

For peer review only

ABSTRACT

Introduction: Strict imaging surveillance protocols to detect complications following Endovascular Aneurysm Repair (EVAR) are common practice. However, controversy exists as to whether all EVAR patients need intense surveillance. The 2019 European Society for Vascular Surgery (ESVS) guidelines for management of abdominal aortic aneurysm (AAA) suggest that patients may be considered for limited follow-up with imaging if classified as 'low risk' for complications based on their initial postoperative imaging. The current study aims to investigate the intervention-free survival and overall survival stratified for patients with and without yearly imaging surveillance.

Methods and analysis: The ODYSSEUS study comprises a national multicentre retrospective cohort study in 17 medical centres. Consecutive patients with an asymptomatic or symptomatic infrarenal AAA who underwent EVAR between January 2007 and January 2012 will be included in this study with follow-up until December 2018. Clinical variables and all follow-up information will be retrieved in extensive data collection from the patient's medical records. In addition, an e-survey was sent to vascular surgeons at the 17 participating centres to gauge their opinions regarding the possibility of safely reducing the frequency of imaging surveillance. Primary endpoints are intervention after EVAR and aneurysm related mortality. The initial estimated sample size is 1997 patients.

Ethics and dissemination: The study has been approved by the Medical Ethics Review Committee of the Amsterdam UMC, location Academic Medical Centre, Amsterdam, the Netherlands. Study findings will be disseminated via presentations at conferences and publications in peer-reviewed journal.

Registration: Netherlands Trial Registry, NL6953 (old: NTR28773). Registered 5 Apr 2018. URL: https://www.trialregister.nl/trial/6953

For peer review only

ARTICLE SUMMARY

Strengths and limitations of this study

- The main strength of this study is that it can accumulate data from large number of patients with long-term follow-up up to 11 years and that it captures all surveillance visits, long-term outcomes and mortality post-EVAR.
- The sample size will be large enough to enable survival and regression analyses in sub-groups of patients.
- The main limitation of the study is due to the nature of retrospective data, it allows only the collection of data that was documented in the patient medical records.

For peer review only

INTRODUCTION

Endovascular Aneurysm Repair (EVAR) of the abdominal aorta has become the primary treatment of patients with an abdominal aortic aneurysm (AAA).[1] Both the Society for Vascular Surgery (SVS) International Guidelines and the instructions for use (IFU) of endograft manufacturers recommend yearly imaging surveillance for all patients after EVAR.[2] However, if the patient is classified as 'low risk' for complications based on initial post-operative imaging, the 2019 European Society for Vascular Surgery (ESVS) guidelines recommend delaying imaging until five years after repair.[3] This movement towards reducing the imaging frequency will benefit patients, medical centres and health care costs.

Imaging surveillance by Computed Tomography Angiography (CTA) may increase the attributable lifetime cancer risk of patients, as well as putting them at risk of developing nephropathy due to contrast exposure. If yearly CTA is replaced by duplex ultrasonography (DUS) patients still experience the burden of additional hospital visits. Moreover, compliance with yearly imaging is suboptimal and non-adherence to yearly imaging does not appear to be associated with poorer outcomes.[4][5]

It has been questioned whether yearly imaging is necessary for all EVAR patients, and if a specific group of patients can be identified for which surveillance intervals can safely be extended, as is suggested by the new guideline.[3] For these reasons, in the Netherlands the Observing a Decade of Yearly Standardised Surveillance in EVAR patients with Ultrasound or CT Scan (ODYSSEUS) study has been designed. In this study of approximately 2000 patients with 6-11 years of follow-up, we aim to determine when, and in which patients, it is safe to deviate from the current annual surveillance protocols.

For peer review only

Background and relevant literature and data

Before initiating the ODYSSEUS study, we conducted a survey among Dutch vascular surgeons to find out if they support the possibility of reducing the frequency of imaging surveillance. In this survey, vascular surgeons reported the main reasons patients did not comply with follow-up visits, i.e. they had forgotten the appointment or were prevented by force majeure. Most physicians estimated that less than 10% of their patients had missed one or more follow-up visits post-EVAR. This might be an overestimation of the true adherence to follow-up visits, as these observations are in contrast with a study reporting that only 43% of patients had complete surveillance.[4]

We also asked participating vascular surgeons to upload their standard post-EVAR protocol to investigate if there were differences between centres in the Netherlands. In all centres imaging took place within the first 3 months after surgery, mostly by CTA. Most centres comply with their own post-EVAR surveillance protocols, which have many commonalities with the SVS and ESVS guidelines. Only one centre utilizes precisely the same post-EVAR surveillance protocol as recommended by the SVS guidelines. Another centre had already reduced follow-up imaging to once every five years, using either CTA or DUS as is stated in the new ESVS guidelines.[3] While vascular surgeons still seem to adhere to their hospital-specific protocol, they do support the need for reducing follow-up by selecting a group of patients for which yearly follow-up can safely be omitted. However, some surgeons indicated that more evidence is needed than is available in the current literature.[6][7]

In studies that have investigated the indications for post-EVAR intervention, it is stated that 61-98% of interventions were necessary because of symptoms and not because of findings at surveillance imaging. This suggests that post-EVAR surveillance protocols provide no benefit to a large group of patients, as complications occur in between surveillance visits.[8][9] Imaging surveillance may even lead to unnecessary interventions and it does not appear to be associated with improved survival.[9][10] We hypothesize that the requirement

For peer review only

for routine imaging for patients at low risk can be reduced. However, novel endovascular devices still require more intensive surveillance as the short- and long-term results of those devices remain undetermined.

For peer review only

STUDY OBJECTIVES

The objective of this study is to evaluate whether imaging surveillance frequency might have been safely reduced in a selected group of EVAR patients, for example in patients with an asymptomatic or symptomatic infrarenal AAA who underwent EVAR and who had no abnormalities on the 3 month postoperative CTA. The clinical course of a large cohort of patients will be evaluated with follow-up ranging between 6 and 11 years. Baseline patient characteristics, aortic anatomy and details of the operation will be derived from the patient's medical record. The first milestone during follow-up is the first postoperative CTA. This scan either shows complications such as endoleaks, malposition or migration of the graft, or the absence thereof. All follow-up visits, imaging studies, as well as all interventions after EVAR and outcomes will be registered. Our hypothesis is that patients with less follow-up will have better outcomes regarding the number of interventions and aneurysm related mortality compared to patients with annual follow-up. Regarding the intervention rates, it is expected that adherence to imaging surveillance may detect more abnormalities triggering re-interventions, which in itself may cause additional complications and perhaps even a decrease in survival rates. We hypothesize that the need for routine imaging for patients with no abnormalities at their initial CTA can be decreased.

For peer review only

METHODS AND ANALYSIS

The study protocol has been designed according to the Standard Protocol Items: Recommendations for Interventional Trials (SPIRIT) statement and the CONsolidated Standards of Reporting Trials (CONSORT).[11,12] The study is registered in The Netherlands National Trial Registry as registration number NL6953**,** available at: https://www.trialregister.nl/trial/6953

General study design

A multicentre retrospective cohort study in 17 medical centres in the Netherlands. Data will be collected from the medical records of all consecutive patients with AAA who underwent EVAR between January 2007 and January 2012. This selection provides a theoretical length of follow-up of 6-11 years on December 2018. Patients will be divided into three groups: A) patients without abnormalities at their first postoperative CTA with yearly imaging surveillance, B) patients without abnormalities at their first postoperative CTA without yearly imaging surveillance and C) patients with abnormalities at their first postoperative CTA (Figure 1). This retrospective design has the advantage of collecting long term follow-up data. The Dutch Dream trial found that the number of interventions starts to rise 4 years after EVAR and the long-term results of the EVAR-1 trial show that EVAR has an early survival benefit but inferior late survival compared with open surgical repair.[13,14] This is in contrast to the recently published long-term results of the OVER trial in which no difference was observed between EVAR and OSR in the primary outcome of all-cause mortality.[15] Hence, a prospective study would take approximately 8 to 10 years to gather enough patients with adequate follow-up.

The standard of care is defined by the current guidelines and instructions for use (IFU). The usual follow-up schedule in the IFU is: CTA and abdominal X-ray at 30 days, 6 and 12 months and yearly thereafter. The 2019 ESVS guidelines recommend a CTA 30 days after

For peer review only

EVAR. If there is adequate seal and no endoleak patients are classified as low risk and CTA follow-up may take place 5 years later. If there is an inadequate seal and endoleak type I/III patients could be either evaluated for re-intervention or if sac shrinkage occurs yearly DUS is recommended. In the 2018 SVS guideline CTA at one and 12 months is recommended and if neither endoleak nor sac enlargement is documented, DUS is suggested for annual postoperative surveillance. In our study design the definition of compliance is undergoing imaging surveillance every 16 months since patients in most centres will be rescheduled if they missed their annual follow-up visit. Device-specific complications after EVAR will also be examined.

Study population

Patients eligible for this retrospective study are all adults who underwent elective EVAR for asymptomatic or symptomatic infrarenal AAA between January 2007 and January 2012. Table 1 gives a more detailed overview of the inclusion and exclusion criteria.

Patient and public involvement

No patients were involved in the research design and conception of this research study.

Date range of the study

Data will be extracted from patient medical records retrospectively and entered into a database with data validation from December 2018 until June 2020. At first two researchers will extract data together to standardize data extraction. Next, to further improve the validity of the data two researchers will independently extract data and enter it into the secured data base. Disagreements will be noted and resolved by discussion and if necessary by asking another co-author to act as an arbiter.

Subject selection

For peer review only

A retrospective cohort study of consecutive patients treated at 17 vascular centres is to be performed. All patients are eligible and the opt-out procedure will be used to allow patients to object to participation within four weeks, which is in accordance with the Dutch Code of Civil Procedure. The Medical Ethics Review Committee of the Amsterdam UMC, location Academic Medical Centre, Amsterdam, has confirmed that the Medical Research Involving Human Subjects Act (WMO) does not apply to our study. This study is conducted according to the General Data Protection Regulation (AVG 2016) and the Medical Treatment Agreement Act (WGBO).

Data sources

Paper or electronic medical records are used in order to identify participants who match study-defined criteria.

Primary and secondary endpoints

Main study endpoint:

- The number of patients with an intervention and aneurysm related mortality classified for patients with and without yearly imaging surveillance.

Secondary study endpoints:

- Date, type, indication and outcome of all postoperative imaging during follow-up.
- Type I, type II, type II and type IV endoleak, graft or outflow (iliac) occlusion, endograft infection detected by postoperative imaging, if present.
- Date and type of intervention during follow-up, if present.
- Date of aneurysm rupture during follow-up, if present.
- Date of death during follow-up, if present.
- Costs of all EVAR related imaging and outpatient clinic visits.

For peer review only

Study procedures

The primary outcomes of this study are interventions and aneurysm related mortality for patients who had a normal initial postoperative CTA and who do adhere to our definition of yearly imaging surveillance over a 6 to 11-year follow-up period, compared to those who do not adhere to our definition.

Interventions are EVAR-related interventions defined by the SVS reporting standards as postoperative adjunctive manoeuvres.[16][17] Interventions for wound complications at the access site are not included, since these are detectable without the use of imaging.

Date of death during follow-up, if applicable, will be obtained from patient medical records and verified by the Dutch municipal personal records database (GBA).

Details of all surveillance imaging are obtained from patient medical records and radiology reports. The time between imaging appointments is calculated to determine whether patients adhere to this studies definition of with yearly imaging surveillance, i.e. within every 16 months.

Date, type, indication and outcome of all postoperative imaging during follow-up are obtained from patient medical records, specifically imaging order forms and radiology reports. A normal initial postoperative CT scan is defined as a CT scan which shows no endoleaks, endograft migration (>10 mm), kinking or obstruction. All imaging outcomes are based on the report compiled by radiologists. These reports will not be re-evaluated by an independent radiologist, since we want to base our outcomes on real life data.

Secondary outcomes are all-cause mortality, type I, type II, type III and type IV endoleak, graft or outflow (iliac) occlusion, aneurysm rupture and endograft infection. This is also obtained from patient medical records, specifically radiology reports and Dutch municipal personal records database (GBA).

Date of aneurysm rupture is obtained from patient medical records, specifically operative reports, radiology reports and progress notes.

For peer review only

Costs of all EVAR-related imaging and outpatient clinic visits will be calculated per patient. Cost is defined as volume times price. Prices from the "Cost manual of the Dutch Health Care Institute" will be used. Costs for the patients will also be included. The quality-adjusted life years (QALYs), a generic measure of disease burden including both the quality and quantity of life, cannot be calculated with this retrospective design.

Sample size and power

Sample size calculation for this study is based on an expected difference of 7% in the proportions of patients not requiring interventions after 7 years between patients undergoing yearly standardised imaging surveillance (75% intervention-free rate [18]) versus those s not undergoing standardised imaging surveillance(82% intervention-free rate[19]). To detect this difference with 90% power and a 0.05 significance level, 719 patients per group are required and 1438 in total. To correct for the fact that the first CTA of approximately 20% of patients is abnormal, 1798 patients (1438/0.8) are needed.[20][21] In addition, we expect incomplete data in 10% of the patients which results in a total number of 1997 patients(1798/0.9).. With this sample size we can also detect a 3% difference in aneurysm related mortality with statistical significance. We chose a one-sided significance level (non-inferiority) of 0.05 and for standard proportion a 95% non-aneurysm related mortality and thus 5% freedom from aneurysm-related mortality after 7 years: 0.95. For equivalence limit difference, we chose an acceptable difference between groups of 3%, in which if differences in aneurysm related mortality equals 3%, they are considered non-inferior. Test-expected proportion is then equal to the standard proportion 0.97. Thus, the expected difference is 0, calculated with a power of 80%. This results in 653 patients per group and 1306 patients in total. Since the first CTA of approximately 20% of patients is abnormal, 1632 patients (1306/0.8) are needed. In addition, we expect incomplete data in

For peer review only

10% of the patients which results in a minimum of, 1813 patients (1632/0.9) are needed in total.

Statistical methods

Differences in baseline characteristics between patients undergoing or not undergoing yearly standardised imaging surveillance by either CTA or DUS, will be analysed using the Chi-square or Fisher's exact test for categorical variables and the student's t-test or Mann Whitney test for continuous variables, if appropriate.

The primary endpoints, i.e. survival and freedom from intervention will be estimated by Kaplan-Meier survival analysis and differences between groups will be assessed with the log-rank test.

Secondary endpoints such as freedom from aneurysm rupture between patients with and without yearly standardised imaging surveillance will be estimated by Kaplan-Meier survival analysis, and differences between these groups will be calculated with the log-rank test.

Multivariable cox regression analysis will be used to determine survival and the freedom of intervention corrected for age, gender, AAA diameter, ASA classification, neck length, neck angulation and type of endograft. The association between postoperative intervention and the following covariates will be investigated with the multivariate Cox-regression analysis:

- age
- gender
- AAA diameter
- ASA classification
- neck length (>15mm)
- neck angulation (>60°)

For peer review only

- type of endograft
- initial postoperative CTA

All statistical analyses will be done with SPSS software (IBM, version 25). The level for statistical significance is set at a p-value < 0.05. The proportion of missing data will be displayed. The missing values will be imputed by multiple imputation techniques if this does not exceed 10-15% and conduct a sensitivity analysis to investigate the effect of the missing data on the results of the analysis. If missing data on outcome variables exceeds 15% we plan to perform subgroup analysis. **DISCUSSION**

The goal of this study is to evaluate whether a reduction in follow-up visits and imaging and thus costs, in patients operated on for an asymptomatic or symptomatic AAA with EVAR is safe. We hypothesize that there will be less interventions and no difference in aneurysm related mortality in patients with less intensive follow-up. With the results of this study we aim to provide scientific evidence helping vascular surgeons decide whether less vigilant follow-up after EVAR may be considered for patients classified in the low risk group.

The strengths of the ODYSSEUS study are that it can accumulate the data of a large number of consecutive EVAR patients with a theoretical follow-up of 6 to 11 years, and that it captures all surveillance visits, long-term outcomes and mortality post-EVAR. Moreover, 17 medical centres throughout the Netherlands are participating in this study, including university and general hospitals, thereby reducing selection bias.

An e-survey has been sent to all vascular surgeons participating in the ODYSSEUS study. This shows that yearly imaging surveillance is upheld by most vascular surgeons in the Netherlands. In addition, most physicians agree that yearly imaging frequency can be safely reduced in a specific group of EVAR patients. As support for this reduction in frequency is evident in the Netherlands, the next step will be to study the groups for which it will be safe to deviate from the widely accepted surveillance protocols.

Our study is subject to limitations due to the nature of administrative data and its retrospective and observational design. As with all studies using administrative data, it allows only the collection of data that was documented in patient medical records. It is also possible that some patients may have transferred to alternative surveillance protocols in

For peer review only

different medical centres without our knowledge. The study will assess results in 17 medical centres over 11 years, during which time improvements in endograft and in clinical practice has occurred. Attrition bias due to loss to follow up represents a threat to the internal validity of our cohort study. The mentioned e-survey has only been sent to participating vascular surgeons, perhaps surgeons participating in the ODYSSEUS study strongly believe that imaging surveillance frequency can be reduced. This may have provided a biased view of post-EVAR follow-up in the Netherlands. However, most of the high-volume EVAR centres in the Netherlands have been included. Another limitation is that no information is retrieved from patients' medical records about when not to intervene and what the reason was for this decision.

In conclusion, with the ODYSSEUS study we aim to confirm the follow-up protocol of the recent ESVS guideline delaying imaging after 5 years if classified in the low risk group and therefore aim to investigate the intervention-free-survival and aneurysm related mortality for patients with and without yearly imaging surveillance.

For peer review only

ETHICS AND DISSEMINATION

The Medical Ethics Review Committee of the Academic Medical Centre, Amsterdam, has reviewed and approved our study protocol version 1.6 dated 26 March 2018. The study is being conducted according to the principles of the Declaration of Helsinki in the current version of Fortaleza, Brazil (2013). Principles of good clinical practice will be respected. Study participation is voluntary. We aim to produce high-impact peer-reviewed publications of the results of the study and present our findings at national and international conferences. The members of the project group of this study will be involved in preparing manuscript drafts and abstract among any other publications arising from the study. The Netherlands Organization for Health Research and Development demands us to stay in close cooperation with the patients association ('Harteraad'). The results of this study will be shared with the members of the patients association via multiple modalities.

For peer review only

STUDY ADMINISTRATION

Data management

All data obtained during the course of the study are considered to be confidential and will not be distributed to third parties. Patient data are stored anonymously under a code. Only the principal investigator or researchers authorised by the principal investigator have access to the key file.

Informed consent

For this retrospective study no informed consent is required. Patients are informed by the researchers that their patient information will be used for research. Patients are able to opt-out of their information being used by returning the opt-out form attached to the patient information letter via email or stamped return envelopes.

Funding statement

This study is funded by the AMC Foundation and with financial support of the Netherlands Organization for Health Research and Development (ZonMw; grant 843004119). The duration of the study is from July 15, 2018 to July 15, 2021. The AMC Foundation nor ZonMw is involved in the study design, writing of the manuscript or the decision to submit the manuscript for publication.

Conflict of interest

The authors declare that they have no competing interests.

Author contributions

For peer review only

Conception and design of study: A.C.M. Geraedts, S.M.L. de Mik, D.T. Ubbink, M.W.J. Koelemay, R. Balm.

Drafting the manuscript: A.C.M. Geraedts, S.M.L. de Mik.

Revising the manuscript critically for important intellectual content: D.T. Ubbink, M.W.J. Koelemay, R. Balm.

Approval of the version of the manuscript to be published: A.C.M. Geraedts, S.M.L. de Mik, D.T. Ubbink, M.W.J. Koelemay, R. Balm.

Agreement to be accountable for all aspects of the work: A.C.M. Geraedts, S.M.L. de Mik, D.T. Ubbink, M.W.J. Koelemay, R. Balm.

Data statement

Castor EDC, the Netherlands will be used for data collection and will be managed by qualified personnel. The data output can be opened in SPSS. Data is published via an DOI-code that will be requested via Figshare. Storage of the data under the DOI-code is one of the requirements of ZonMw.

For peer review only

REFERENCES

1.The EVAR trial participants. Comparison of endo vascular aneurysm repair with open repair in patients with abdominal aortic aneurysm (EVAR trial 1), 30-day operative mortality results: randomized controlled trial. Lancet 2004; 364: 843–48.

2. Chaikof EL, Brewster DC, Dalman RL, et al. SVS Practice guidelines for the care of patients with an abdominal aortic aneurysm: executive summary. J Vasc Surg 2018;67(1):2-77..

3. Wanhainen A, Verzini F, van Herzeele I, et al. European Society for Vascular Surgery (ESVS) 2019 Clinical Practice Guidelines on the Management of Abdominal Aorto-Iliac Artery Aneurysms. Eur J Vasc Endovasc Surg. 2019 Jan;57(1):8-93.

4. Garg T, Baker LC, Mell MW. Postoperative Surveillance and long-term outcomes after endovascular aneurysm repair among Medicare beneficiaries. JAMA Surg 2015;150:957-63.

5. Kret MR, Azarbal AF, Mitchell EL, et al. Compliance with long-term surveillance recommendations following endovascular aneurysm repair or type B aortic dissection. J Vasc Surg 2013 Jul;58(1):25-31.

6. Baderkhan H, Haller O, Wanhainen A, Björck M, Mani K. Follow-up after endovascular aortic aneurysm repair can be stratified based on first postoperative imaging. Br J Surg 2018;105:709-18

7. Bastos Goncalves F, Baderkhan H, Verhagen HJ, Wanhainen A, Björck M, Stolker RJ, et al. Early sac shrinkage predicts a low risk of late complications after endovascular aortic aneurysm repair. Br J Surg 2014;101:802-10.

8. Karthikesalingam A, Holt PJ, Hinchliffe RJ, et al. Risk of reintervention after endovascular aortic aneurysm repair. Br J Surg 2010;97(5):657-63.

9. Dias NV, Riva L, Ivancev K, et al. Is there a benefit of frequent CT follow-up after EVAR? Eur J Vasc Endovasc Surg 2009;37(4):425-30.

10. De Mik SML, Geraedts ACM, Ubbink DT, et al. Effect of imaging surveillance after Endovascular Aneurysm Repair on reinterventions and mortality: a systematic review and meta-analysis. J Endovasc Ther. 2019;26(4):531-541.

For peer review only

11.Chan AW, Tetzlaff JM, Gotzsche PC, Altman DG, Mann H, Berlin JA, et al. SPIRIT 2013 explanation and elaboration: guidance for protocols of clinical trials. BMJ. 2013;346.

12. Campbell MK, Piaggio G, Elbourne DR, Altman DG, Group C. Consort 2010 statement: extension to cluster randomised trials. BMJ. 2012;345.

13. De Bruin Jorg L, Baas Annette F, Buth Jaap, et al, for the DREAM Study Group. Long-term outcome of open or endovascular repair of abdominal aortic aneurysm. N Engl J Med 2010;362:1881e9.

14. Patel R, Powell JT, Sweeting MJ, et al. The UK EndoVascular Aneurysm Repair (EVAR) randomised controlled trials: long-term follow-up and cost-effectiveness analysis. Health Technol Assess. 2018 Jan;22(5):1-132.

15. Lederle FA, Kyriakides TC, Stroupe, KT, et al. Open versus endovascular repair of abdominal aortic aneurysm. N Engl J Med 2019;380(22):2126-2135.

16. Chaikof EL, Blankensteijn JD, Harris PL, et al. Reporting standards for endovascular aortic aneurysm repair. J Vasc Surg. 2002;35(5):1048-60.

17. Alonso J, Bartlett SJ, Rose M, et al. The case for an international patient-reported outcome measurement information system (PROMIS®) initiative. Health Qual life Outcomes 11:210, 2013.

18. Wu CY, Chen H, Gallagher KA, et al. Predictors of compliance with surveillance after endovascular aneurysm repair and comparative survival outcomes. J Vasc Surg. 2015;62(1):27-35.

19. Conrad MF, Adams AB, Guest JM, et al. Secondary intervention after endovascular abdominal aortic aneurysm repair. Ann Surg. 2009;250(3):383-9.

20. Patel MS, Carpenter JP. The value of the initial post-EVAR computed tomography angiography scan in predicting future secondary procedures using the Powerlink stent graft. J Vasc Surg. 2010;52(5):1135-9.

For peer review only

21. Troutman DA, Chaudry M, Dougherty MJ, et al. Endovascular aortic aneurysm repair surveillance may not be necessary for the first 3 years after an initially normal duplex postoperative study. J Vasc Surg. 2014;60(3):558-62.

For peer review only

Tables

Table 1

Retrospective cohort

Inclusion criteria	Exclusion criteria
Age ≥ 18 years<br>Patient with an (a)asymptomatic infrarenal abdominal aortic aneurysm<br>EVAR between January 2007 and January 2012<br>Patients with an initial postoperative CTA within 90 days after EVAR	Connective tissue disease<br>Patients who objected to their retrospective data being used

For peer review only

Figures

Figure 1. Patient subgroups

For peer review only

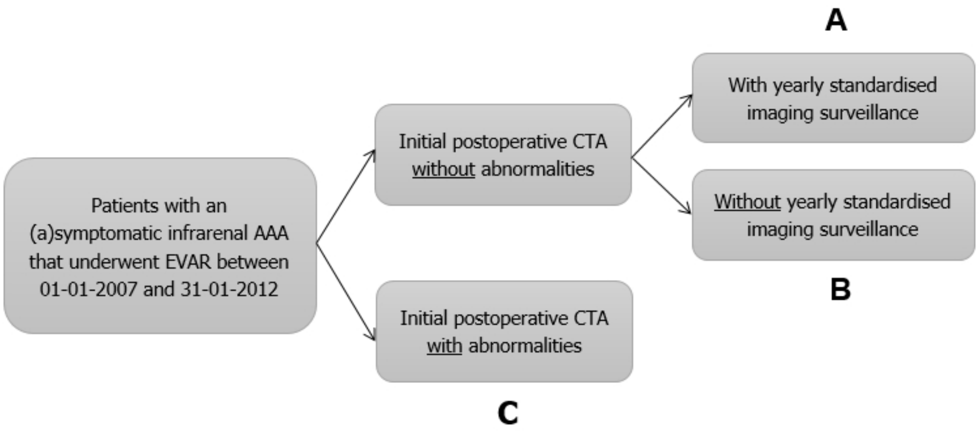

Figure 1. Patient subgroups

STROBE Statement—checklist of items that should be included in reports of observational studies

	Item No	Recommendation	Page No
**Title and abstract**	1	(*a*) Indicate the study's design with a commonly used term in the title or the abstract	1
		(*b*) Provide in the abstract an informative and balanced summary of what was done and what was found	3
**Introduction**			
Background/rationale	2	Explain the scientific background and rationale for the investigation being reported	4
Objectives	3	State specific objectives, including any prespecified hypotheses	7
**Methods**			
Study design	4	Present key elements of study design early in the paper	9
Setting	5	Describe the setting, locations, and relevant dates, including periods of recruitment, exposure, follow-up, and data collection	10-12
Participants	6	(*a*) *Cohort study*—Give the eligibility criteria, and the sources and methods of selection of participants. Describe methods of follow-up *Case-control study*—Give the eligibility criteria, and the sources and methods of case ascertainment and control selection. Give the rationale for the choice of cases and controls *Cross-sectional study*—Give the eligibility criteria, and the sources and methods of selection of participants	9,10
		(*b*) *Cohort study*—For matched studies, give matching criteria and number of exposed and unexposed *Case-control study*—For matched studies, give matching criteria and the number of controls per case	n.a.
Variables	7	Clearly define all outcomes, exposures, predictors, potential confounders, and effect modifiers. Give diagnostic criteria, if applicable	11,12
Data sources/ measurement	8*	For each variable of interest, give sources of data and details of methods of assessment (measurement). Describe comparability of assessment methods if there is more than one group	12,13
Bias	9	Describe any efforts to address potential sources of bias	16,17
Study size	10	Explain how the study size was arrived at	13
Quantitative variables	11	Explain how quantitative variables were handled in the analyses. If applicable, describe which groupings were chosen and why	14
Statistical methods	12	(*a*) Describe all statistical methods, including those used to control for confounding	14
		(*b*) Describe any methods used to examine subgroups and interactions	14
		(*c*) Explain how missing data were addressed	13,14
		(*d*) *Cohort study*—If applicable, explain how loss to follow-up was addressed *Case-control study*—If applicable, explain how matching of cases and controls was addressed *Cross-sectional study*—If applicable, describe analytical methods taking account of sampling strategy	16
		(*e*) Describe any sensitivity analyses	n.a.

Continued on next page

For peer review only - http://bmjopen.bmj.com/site/about/guidelines.xhtml

**Results**			
Participants	13*	(a) Report numbers of individuals at each stage of study—eg numbers potentially eligible, examined for eligibility, confirmed eligible, included in the study, completing follow-up, and analysed	N.a.
		(b) Give reasons for non-participation at each stage	N.a.
		(c) Consider use of a flow diagram	N.a.
Descriptive data	14*	(a) Give characteristics of study participants (eg demographic, clinical, social) and information on exposures and potential confounders	N.a.
		(b) Indicate number of participants with missing data for each variable of interest	N.a.
		(c) *Cohort study*—Summarise follow-up time (eg, average and total amount)	N.a.
Outcome data	15*	*Cohort study*—Report numbers of outcome events or summary measures over time	N.a.
		Case-control study—Report numbers in each exposure category, or summary measures of exposure	N.a.
		Cross-sectional study—Report numbers of outcome events or summary measures	N.a.
Main results	16	(*a*) Give unadjusted estimates and, if applicable, confounder-adjusted estimates and their precision (eg, 95% confidence interval). Make clear which confounders were adjusted for and why they were included	N.a.
		(*b*) Report category boundaries when continuous variables were categorized	N.a.
		(*c*) If relevant, consider translating estimates of relative risk into absolute risk for a meaningful time period	N.a.
Other analyses	17	Report other analyses done—eg analyses of subgroups and interactions, and sensitivity analyses	N.a.
**Discussion**			
Key results	18	Summarise key results with reference to study objectives	16
Limitations	19	Discuss limitations of the study, taking into account sources of potential bias or imprecision. Discuss both direction and magnitude of any potential bias	16
Interpretation	20	Give a cautious overall interpretation of results considering objectives, limitations, multiplicity of analyses, results from similar studies, and other relevant evidence	16,17
Generalisability	21	Discuss the generalisability (external validity) of the study results	16,17
**Other information**			
Funding	22	Give the source of funding and the role of the funders for the present study and, if applicable, for the original study on which the present article is based	18

*Give information separately for cases and controls in case-control studies and, if applicable, for exposed and unexposed groups in cohort and cross-sectional studies.

Note: An Explanation and Elaboration article discusses each checklist item and gives methodological background and published examples of transparent reporting. The STROBE checklist is best used in conjunction with this article (freely available on the Web sites of PLoS Medicine at http://www.plosmedicine.org/, Annals of Internal Medicine at http://www.annals.org/, and Epidemiology at http://www.epidem.com/). Information on the STROBE Initiative is available at www.strobe-statement.org.

For peer review only
